# Functional impact of Nth like DNA glycosylase 1 on mitochondrial dynamics

**DOI:** 10.1093/nar/gkag876

**Published:** 2026-09-15

**Authors:** Lisa Hubers, Alexander Myhr Skjetne, Luisa Luna, Yohan Lefol, Solveig Osnes Lund, Ane Marit Wågbø, Xavier Renaudin, Annikka Polster, Zoe J da Silva, Anders Knoph Berg-Eriksen, Francisco Jose Naranjo-Galindo, Anna Campalans, Torkild Visnes, Hilde Loge Nilsen, Nicola Pietro Montaldo

**Affiliations:** Department of Microbiology, Oslo University Hospital and University of Oslo, 0424 Oslo, Norway; Department of Microbiology, Oslo University Hospital and University of Oslo, 0424 Oslo, Norway; Department of Microbiology, Oslo University Hospital and University of Oslo, 0424 Oslo, Norway; Department of Microbiology, Oslo University Hospital and University of Oslo, 0424 Oslo, Norway; Cresco Centre for Embryology and Healthy Development, Institute of Clinical Medicine, University of Oslo, 0318 Oslo, Norway; Department of Microbiology, Oslo University Hospital and University of Oslo, 0424 Oslo, Norway; Department of Biotechnology and Nanomedicine, SINTEF Industry, 7034 Trondheim, Norway; Université Paris-Saclay, iRCM/IBFJ, CEA, Genetic Stability, Stem Cells and Radiation, F-92260 Fontenay-aux-Roses, France; Université Paris Cité, iRCM/IBFJ, CEA, Genetic Stability, Stem Cells and Radiation, F-92260 Fontenay-aux-Roses, France; Department of Microbiology, Oslo University Hospital and University of Oslo, 0424 Oslo, Norway; Division of Systems and Synthetic Biology, Department of Life Sciences, Chalmers University of Technology, 41296 Gothenburg, Sweden; Department of Microbiology, Oslo University Hospital and University of Oslo, 0424 Oslo, Norway; Department of Human Genetics, Leiden University Medical Center, 2300 RC Leiden, The Netherlands; Department of Microbiology, Oslo University Hospital and University of Oslo, 0424 Oslo, Norway; Department of Microbiology, Oslo University Hospital and University of Oslo, 0424 Oslo, Norway; Université Paris-Saclay, iRCM/IBFJ, CEA, Genetic Stability, Stem Cells and Radiation, F-92260 Fontenay-aux-Roses, France; Université Paris Cité, iRCM/IBFJ, CEA, Genetic Stability, Stem Cells and Radiation, F-92260 Fontenay-aux-Roses, France; Department of Biotechnology and Nanomedicine, SINTEF Industry, 7034 Trondheim, Norway; Department of Microbiology, Oslo University Hospital and University of Oslo, 0424 Oslo, Norway; Cresco Centre for Embryology and Healthy Development, Institute of Clinical Medicine, University of Oslo, 0318 Oslo, Norway; Department of Microbiology, Oslo University Hospital and University of Oslo, 0424 Oslo, Norway; Cresco Centre for Embryology and Healthy Development, Institute of Clinical Medicine, University of Oslo, 0318 Oslo, Norway

## Abstract

Nth like DNA glycosylase 1 (NTHL1), a key base excision repair enzyme, has long been considered essential for nuclear and mitochondrial genome integrity. Combining *in vitro* biochemical assays, *in cellulo* molecular biology, and bioinformatic analyses, we investigated how NTHL1 loss affects mitochondrial DNA (mtDNA) stability and mitochondrial function. Contrary to the conventional view that mtDNA damage is solely detrimental, we find that NTHL1 loss confers a beneficial, mitochondria-initiated phenotype in human cells. Despite accumulating mtDNA lesions, NTHL1 loss unexpectedly increases mtDNA copy number, elevates oxidative phosphorylation protein levels, and enhances mitochondrial respiration. *NTHL1*^−/−^ cells also show increased mitochondrial mass and higher levels of the biogenesis regulator PGC1α and the fusion protein OPA1, indicating an adaptive response that boosts mitochondrial function and capacity. Consequently, *NTHL1^−/−^* cells exhibit resistance to mitochondrial stress, accompanied by increased eIF2α phosphorylation and reduced MYC levels, converging on a broader transcriptional adaptive program. This phenotype depends on mitochondrial NTHL1 and reactive oxygen species (ROS) signaling, since treatment with ROS scavengers or mitochondria-specific reintroduction of NTHL1 rescues it. Together, these findings position NTHL1 as a key modulator of mtDNA stability and mitochondrial function, revealing that loss of this DNA repair enzyme shifts cellular metabolism toward a stress-adaptive state and enhances resilience to oxidative stress.

## Graphical Abstract

**Graphical Abstract:**
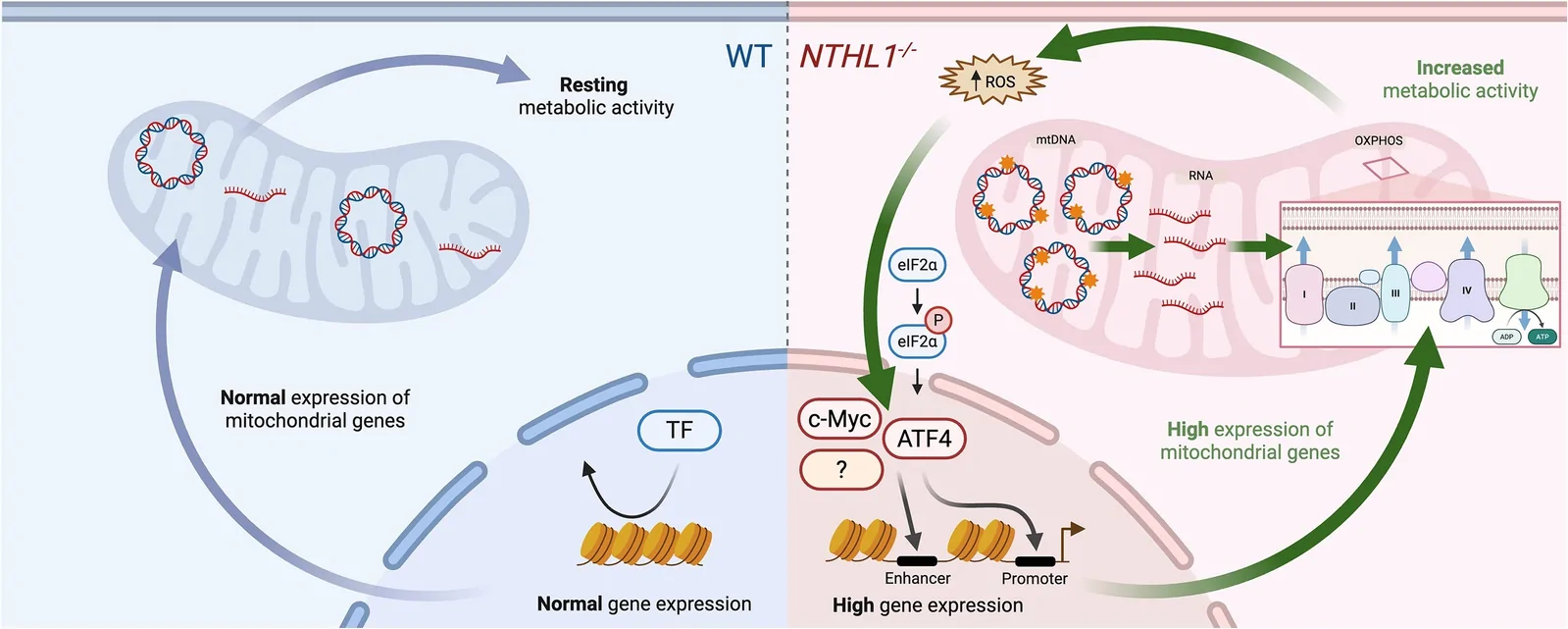

## Introduction

Mitochondria are key organelles that support cellular energy production, metabolism, and signaling. Mitochondrial DNA (mtDNA) encodes 37 essential genes necessary for mitochondrial function and cellular signaling, including critical components of the oxidative phosphorylation (OXPHOS) system, which is indispensable for cellular respiration and aerobic metabolism [1–3]. Mutations or defects in mtDNA, along with alterations in nuclear genes that regulate its maintenance, are linked to mitochondrial disorders in both children and adults [4, 5]. mtDNA is particularly vulnerable to damage due to its proximity to the electron transport chain (ETC) and the lack of protective histones, making it more exposed to reactive oxygen species (ROS) generated during cellular respiration. Its integrity is preserved through multiple mechanisms, including localized antioxidant systems, DNA repair, mtDNA turnover, copy number redundancy, as well as mitochondrial quality control processes such as fission, fusion, and mitophagy [3, 6].

DNA base lesions occur frequently in cells due to exposure to both endogenous and exogenous DNA-damaging agents [7]. Among the various forms of damage, oxidized bases are particularly detrimental, necessitating efficient repair mechanisms to maintain genomic integrity. Base excision repair (BER) is the primary pathway for repair of such DNA lesions [8] and serves as the predominant repair system in mitochondria, especially given the limited presence of other DNA repair mechanisms within this organelle [3]. The BER pathway is initiated by DNA glycosylases, a family of enzymes that recognizes and subsequently excises damaged bases. Bifunctional DNA glycosylases also have the ability to incise the DNA backbone to generate a single-stranded break that is subsequently processed by apurinic/apyrimidinic (AP) endonuclease 1 (APE1). Finally, DNA polymerase β catalyzes the insertion of the missing nucleotides, while DNA ligase III/XRCC1 seals the nick. Various DNA glycosylases have been identified to target specific types of DNA damage, such as 8-oxoguanine, recognized by 8-Oxoguanine DNA Glycosylase 1 (OGG1), and uracil, recognized by Uracil-DNA Glycosylase (UNG).

The human bifunctional Nth like DNA glycosylase 1 NTHL1 (also referred to as hNTHL1, NTH1, or hNTH1) is the evolutionarily conserved homolog of bacterial Endonuclease III (Nth), and excises oxidized pyrimidines like 5-hydroxyuracil (5-ohU) and thymine glycol (Tg) [9]. Recent research suggests a complex interplay between BER, oxidative stress, and mitochondrial dysfunction in neurodegeneration and cancer [10–13]. Specifically, BER activity has been linked to age-dependent accumulation of single-stranded DNA breaks, contributing to pathology in *Caenorhabditis elegans *models of Parkinson’s disease (PD) expressing human α-synuclein [10], as well as Alzheimer’s disease models expressing a pro-aggregant Tau [14].

In these disease models, the *C. elegans* ortholog NTH-1 generates strand breaks during physiological aging, and its loss is associated with a protective phenotype characterized by reduced proteotoxicity and activation of cellular defenses that improve overall health as well as cognitive function [10, 14]. Similarly, NTHL1 overexpression causes genomic instability and early cellular hallmarks of cancer [12, 13]. At the same time, biallelic inactivating variants in the *NTHL1* gene cause NTHL1 tumor syndrome characterized by increased lifetime risk for colorectal cancer [15–17]. Despite these observations, mechanistic insight into the importance of NTHL1 in the context of mammalian aging, neurodegeneration, and cancer remains limited. In particular, the contribution of the mitochondrial NTHL1 function to cellular health is poorly described.

mtDNA copy number (mtDNA-CN) is typically associated with energy production and mitochondrial membrane potential [18]. mtDNA-CN declines during aging, with lower levels being associated with cognitive and physical decline, as well as higher risk of mortality [19–21]. Reductions in mtDNA-CN have been implicated in the progression of PD [22, 23] and could serve as a valuable prognostic marker for disease advancement. mtDNA-CN alterations have also been observed across various cancers [24], with both increases and decreases reported in primary human cancers, potentially contributing to cancer pathogenesis and progression [25]. The exact mechanisms underlying these alterations remain poorly understood, particularly the role of DNA repair pathways and specific DNA lesions in regulating mtDNA-CN. Further investigation is needed to clarify these relationships and their impact on neurodegeneration and cancer development.

In this study, we integrate *in vitro* biochemical assays, *in cellulo* molecular biology experiments, and bioinformatic analyses to unveil the impact of NTHL1 on mtDNA and mitochondrial function. RNA sequencing of CRISPR/Cas9-generated *NTHL1^−/−^* cell lines revealed altered expression of mitochondrial-related genes, particularly those involved in oxidoreductase activity and the ETC, suggesting regulation in key mitochondrial functions such as energy production.

This reprogramming correlated with increased mtDNA-CN, elevated mtDNA damage, and reduced binding of mitochondrial transcription factor A (TFAM) to mtDNA, consistent with defective mtDNA packaging. Respirometry assays showed enhanced mitochondrial respiration in *NTHL1^−/−^* cells, supported by increased mitochondrial mass, as confirmed by immunofluorescence (IF) and transmission electron microscopy (TEM). Notably, *NTHL1^−/−^* cells exhibited greater resistance to mitochondrial stress induced by the complex I inhibitor 1-methyl-4-phenylpyridinium (MPP^+^), suggesting a mitochondrial-specific stress response. This resilience was linked to activation of the integrated stress response (ISR), characterized by increased phosphorylation of eIF2α, a key regulator of mitochondrial function and stress adaptation.

These findings suggest that NTHL1 loss triggers a compensatory response to mtDNA damage, enhancing mitochondrial function through the activation of adaptive stress response pathways to preserve cellular survival and function under oxidative stress.

## Materials and methods

### Cell lines and cell culture

Human embryonic kidney cells (HEK293, ATCC, CRL-1573) and human osteosarcoma cells (U2OS) were cultured at 37°C under a 5% CO_2_ atmosphere using Dulbecco’s Modified Eagle Medium (DMEM) high glucose (Gibco, 11965092) supplemented with 10% fetal bovine serum (FBS) (Merck, F7524) and 1% penicillin/streptomycin (P/S) (Fisher Scientific, 15140130).

Near-haploid HAP1 cell lines, derived from the KBM-7 cell line, were cultured at 37°C under the same conditions using Iscove’s Modified Dulbecco’s Medium (Gibco, 12440061) supplemented with 10% FBS and 1% P/S. The HAP1 cell lines included parental clones wild-type (WT) 1.1, WT 1.3, as well as NTHL1 knockout (KO) clones 10 and 11 (HZGHC001540c010 and HZGHC001540c011, Horizon Discovery, PerkinElmer).

### Generation of HEK293 knockout cell lines

Single guide RNA (sgRNA) targeting *NTHL1* gene and potential off targets were designed using the CRISPOR CRISPR Design tool, and potential off-targets were identified through its predictive algorithm [26]. Alt-R® CRISPR/Cas9 tracrRNA ATTO™550 and Guide (crRNA) were combined in a 1:1 ratio and annealed by heating at 95°C for 5 min, followed by a slow cooling process at room temperature (RT). One micromolar of the resulting sgRNA was mixed with 1 μM Alt-R® S.p. Cas9 Nuclease V3 (IDT) in Opti-MEM and delivered to HEK293 WT using Lipofectamine™ 3000 Transfection Reagent (Invitrogen™). Upon transfection, cells were seeded as single clones into a 96-well plate via serial dilution. Insertion-deletion (Indel) mutations and loss of target protein expression were verified in clonal KO cell lines through Indel-specific polymerase chain reaction (PCR), Sanger sequencing, and immunoblot analysis, respectively. Confirmed HEK293 *NTHL1^−/−^* clones 1, 3, and 15 were here referred to as *NTHL1^−/−^* 1, 2, and 3, respectively. Top-exonic off-target sites with a maximum of three base pair differences from the sgRNA were evaluated in the selected clones via Indel-specific PCR and Sanger sequencing. SgRNA sequences are listed in Table 1.

**Table 1.** List of primers and sequences

Target	Fw/Rv	Sequence (5′–3′)	Assay
**RT-qPCR**			
MT-ND6	Fw	CCCCATGCCTCAGGATACTC	RT-qPCR cDNA
	Rv	TTGTTAGCGGTGTGGTCGG	RT-qPCR cDNA
MT-CYB	Fw	ACCCCCTAGGAATCACCTCC	RT-qPCR cDNA
	Rv	GCCTAGGAGGTCTGGTGAGA	RT-qPCR cDNA
B2M(housekeeping)	Fw	AGGCTATCCAGCGTACTCCA	RT-qPCR cDNA
	Rv	TGTCGGATGGATGAAACCCA	RT-qPCR cDNA
COX4I1	Fw	TTGCTGAGATGAACAGGGGC	RT-qPCR cDNA
	Rv	ATGATAACGAGCGCGGTGAA	RT-qPCR cDNA
COX5B	Fw	CTGCAAAGAAGGGACTGGACC	RT-qPCR cDNA
	Rv	CTTGTTGGAGATGGAGGGGAC	RT-qPCR cDNA
NDUFS6	Fw	GCACACTGGCCAGGTTTATG	RT-qPCR cDNA
	Rv	GCTATCACCCGAGTCTCCAC	RT-qPCR cDNA
HCCS	Fw	CTGCAGAGTGTCCTTGTGGT	RT-qPCR cDNA
	Rv	AGGCAACTCATACCCCATCC	RT-qPCR cDNA
APOOL	Fw	CTGGGACTGGCCACTTTAGG	RT-qPCR cDNA
	Rv	TGCTCCAAAAATTTGCTGGCT	RT-qPCR cDNA
NFU1	Fw	AATACGGCCAACTGTGCAGG	RT-qPCR cDNA
	Rv	GATTGAACTAGGGCAGCTGGT	RT-qPCR cDNA
MAOA	Fw	GAGGTGGCATTTCAGGACTAT	RT-qPCR cDNA
	Rv	CCACATAAGCTCCACCAACA	RT-qPCR cDNA
ACSM3	Fw	TGTCTGCGAACAGGGACAG	RT-qPCR cDNA
	Rv	TTGGATGCAACAGCGTCTACT	RT-qPCR cDNA
ACSL6	Fw	TGCCGGCAGGAACTCTTAAA	RT-qPCR cDNA
	Rv	CAGGTTGGCTCCGGATGTAG	RT-qPCR cDNA
HMOX1	Fw	AAGACTGCGTTCCTGCTCAA	RT-qPCR cDNA
	Rv	GGGGGCAGAATCTTGCACT	RT-qPCR cDNA
TSPO	Fw	TCAGCCATGGGGTACGGC	RT-qPCR cDNA
	Rv	GTACCAGGCCACGGTAGTG	RT-qPCR cDNA
OXCT1	Fw	GCATTTGCTCCAGAAGACATC	RT-qPCR cDNA
	Rv	TCCCTTACGTCATCTCCAGGT	RT-qPCR cDNA
FASN	Fw	GGACACAGTCACCATCTCGG	RT-qPCR cDNA
	Rv	GCTCCCGGATCACCTTCTTG	RT-qPCR cDNA
NTHL1 isoform 1	Fw	GCAACAGCTGGTCAACATCC	RT-qPCR cDNA
	Rv	CACCTGGTACCTGCGTACCTTT	RT-qPCR cDNA
NTHL1 isoform 2	Fw	CCCCCCCAAAGAGCAAGGT	RT-qPCR cDNA
	Rv	AGCCCACCAAGAGTCCATTG	RT-qPCR cDNA
NTHL1 isoform 3	Fw	GGACAGTGAGAAAGGTACGCA	RT-qPCR cDNA
	Rv	ACTGCAATGCCTGACACAGT	RT-qPCR cDNA
GAPDH(housekeeping)	Fw	CCACATCGCTCAGACACCAT	RT-qPCR cDNA
	Rv	CCAGGCGCCCAATACG	RT-qPCR cDNA
**qPCR**			
MT-CYB	Fw	CCCACATCACTCGAGACGTA	qPCR mtDNA-CN
	Rv	CCGATGTTTCAGGTTTCTGAGTA	qPCR mtDNA-CN
MT-ND1	Fw	TCAACATCGAATACGCCGCA	qPCR mtDNA-CN
	Rv	AGTTGGTCGTAGCGGAATCG	qPCR mtDNA-CN
B2M (housekeeping)	Fw	GCTGGGTAGCTCTAAACAATGTATTCA	qPCR mtDNA-CN
	Rv	CCATGTACTAACAAATGTCTAAAATGGT	qPCR mtDNA-CN
MT-RNR2	Fw	CTTCACCAGTCAAAGCGAACT	RADF
	Rv	TAGCGGCTGCACCATCG	RADF
mtDNA	Fw	TCTAAGCCTCCTTATTCGAGCCGA	Long-amp PCR
	Rv	TTTCATCATGCGGAGATGTTGGATGG	Long-amp PCR
mtDNA heavy-strand promoter	Fw	CATCAATACAACCCCCGCCCA	ChIPqPCR
	Rv	ATTGCTTTGAGGAGGTAAGCTACA	ChIPqPCR
**CRISPR/Cas9**			
NTHL1 SgRNA	–	CTCACTGTCCGAGCCCTCAT AGG	SgRNA + PAM
MAST3 off target	–	CTCACTGTCCGAACGCTCAT	Off target
DCTN1 off target	–	CTCACTGCCAGGGCCCTCAT	Off target
NTHL1_indel_Fw_2	Fw	CTCACTGTCCGAGCCCTC	Sequencing
NTHL1_Fw	Fw	CCAAAAGCCACCGGGTAGAA	PCR and indel PCR
NTHL1_Rv	Rv	AACGAGTGAGGGGTGAGCTA	PCR
NTHL1_Fw_Seq	Fw	TTCCTCATGGCACGGATGTT	Sequencing
NTHL1_Rv_Indel	Rv	CTCACTGTCCGAGCCCTC	Indel
MAST3_Fw	Fw	GCAGGATGTGCGTGGTTTTT	PCR and indel PCR
MAST3_Rv	Rv	ACACGATGGGAACTGTCACC	PCR
MAST3_Fw_Seq	Fw	GGTTTTGCATGCAGTGGGAC	Sequencing
MAST3_Rv_Indel	Rv	CTCACTGTCCGAACGCTC	Indel PCR
DCTN1_Fw	Fw	AGAACACTGCTGGGAAGAGC	PCR and indel PCR
DCTN1_Rv	Rv	GGAGGACTGGGATGGGAGAT	PCR
DCTN1_Fw_Seq	Fw	GCTGGTGCGAGTGATGTCTA	Sequencing
DCTN1_Rv_Indel	Rv	CTCACTGCCAGGGCCCTC	Indel PCR
**Site-directed mutagenesis**			
NTHL1 full-length isoform 1	Fw	ATGACCGCCTTGAGCGCGAG	SDM
	Rv	GGCGATCGCGGCGGC	SDM
NTHL1 isoform 1-Δ21–60	Fw	ATGAGGGCTCGGACAGTGAG	SDM
	Rv	GCCCAGCCCCGGGTC	SDM

### PCR, gel electrophoresis, and Sanger sequencing

Total DNA was extracted using the AllPrep kit (Qiagen, 80004). DNA amplification was performed using GoTaq G2 Master Mix (Promega, M7822) with 0.5 µM forward and reverse primers, in a final volume of 50 µl. The amplification was carried out in a thermal cycler, and the PCR products were resolved on a 1% agarose gel. For indel-specific PCR, the forward primer was designed so that its last base would anneal immediately after the CRISPR-Cas9 cut site. This design ensures that Taq polymerase does not elongate the substrate in most cases of indel mutations, resulting in a significantly reduced or absent amplicon. The absence or presence of an amplicon provides a clear yes/no readout from the gel. For Sanger sequencing, the band corresponding to the expected size was visualized under UV light, excised, and purified using the NucleoSpin Gel and PCR Clean-up kit (Macherey-Nagel, 740609.250). The purified PCR product was then sent for sequencing with a single primer located near the region of interest (ROI). Sanger sequencing chromatograms of PCR-amplified CRISPR target regions were analyzed using Deconvolution of Complex DNA Repair (DECODR) [27] to confirm and characterize biallelic indel mutations at the target site. Primer sequences are listed in Table 1. Kits and reagents are listed in Table 2.

**Table 2.** List of kits and reagents

Reagent or resource	Source	Identifier
Mitochondria isolation kit	Miltenyi	130-094-532
AllPrep DNA/RNA/Protein Mini Kit	Qiagen	80 004
MitoView Green	Biotium	70 054
Mitotracker Red	Invitrogen	M22425
N-acetyl cysteine	Sigma	A9165
XJB-5–131	Sigma	SML2982
Cell fractionation kit	Abcam	109 719
Lipofectamine 2000	Thermo Fisher	11 668 027
Lipofectamine 3000	Thermo Fisher	L3000001
Mitophagy Detection Kit	Dojindo	MD01

### Mitochondria extraction

Mitochondria were isolated from HEK293 cells using the mitochondria isolation kit from Miltenyi Biotec (130-094-532) according to manufacturer’s protocol. Briefly, 1 × 10^7^ cells were collected, resuspended in lysis buffer, and homogenized using a Dounce homogenizer. Anti-TOM22 microbeads were used to magnetically label mitochondria, followed by column-based magnetic separation. Mitochondria were used immediately for downstream analysis. Kits are listed in Table 2.

### Cellular fractionation

5 × 10^6^ HEK293 cells were collected and fractionated using a cell fractionation kit (Abcam, 109719) into cytosolic (C), mitochondrial (M), and nuclear (N) fractions following manufacturer’s protocol. Fractions were subject to immunoblot analysis as described below.

### Plasmid preparation

pCMV6-AC-GFP-NTHL1 was purchased from Origene (RG214598). Using site-directed mutagenesis (SDM), the first 8 amino acids were removed from the N-terminus of NTHL1 to generate isoform 1. NTHL1 isoform 1 was then excised and cloned into pcDNA3-mRFP (Addgene, 13032) using restriction digestion. Amino acids 21–60 were removed via SDM. Primer sequences are listed in Table 1, and plasmids are listed in Table 3.

**Table 3.** List of plasmids

Reagent or resource	Source	Identifier
pCMV6-AC-GFP-NTHL1	Origene	#RG214598
pcDNA3-mRFP	Addgene	#13032
pcDNA3.1-NTH1-FLAG	This study	
pcDNA3-NTHL1 Isoform 1-mRFP (NTHL1-RFP)	This study	
pcDNA3-NTHL1 Isoform 1-Δ21–60-mRFP (NTHL1(Δ21–60)-RFP)	This study	

### Transfection

U2OS cells were transfected with the plasmid pcDNA3.1-NTH1-FLAG at final concentration of 100 ng/ml using Lipofectamine 2000 (Thermo Fisher, 11668027) according to manufacturer’s instructions. HEK293 cells were transfected using the NTHL1-RFP plasmids, each at a concentration of 200 ng/ml using Lipofectamine 3000 (Thermo Fisher, L3000001) following manufacturer’s protocol, and incubated for 72 h in 5% CO_2_ and 37°C.

### Library construction, quality control, and sequencing

Total RNA was extracted from four biological replicates of HEK293 WT and *NTHL1^−/−^* clones using AllPrep kit (Qiagen, 80004), and DNase I digested (Qiagen, 79254) according to the manufacturer’s protocol. Total RNA was used as the input material for RNA sample preparations. RNA library preparation and sequencing were carried out by Novogene Europe. Ribosomal RNA (rRNA) was removed from total RNA using specific probes. RNA fragmentation was performed using divalent cations at elevated temperatures in the First Strand Synthesis Reaction Buffer. First-strand complementary DNA (cDNA) was synthesized using random hexamer primers and reverse transcriptase. The second strand of cDNA was then synthesized by adding buffer and dNTPs, where dTTP was replaced by dUTP. The synthesized double-stranded cDNA was subjected to end repair, A-tailing, adaptor ligation, and PCR enrichment following fragment selection. To select cDNA fragments ranging from 370 to 420 bp, PCR products were purified using AMPure XP beads, resulting in a strand-specific library. Following library construction, preliminary quantification was performed using Qubit. The size of the inserted fragments was evaluated, and the library’s effective concentration was quantified using real-time qPCR. The qualified libraries were then pooled and sequenced on Illumina platforms according to the required effective concentration and data output. The original fluorescence image files generated by the Illumina platform were converted into short reads (raw data) through base calling. The resulting short reads were stored in FASTQ format [28].

### RNA sequencing processing

Quality control was applied to the raw reads to eliminate sequence artifacts, such as adapter contamination, low-quality nucleotides, and unrecognizable nucleotides (N). Quality control was performed using Fastp (v0.23.1) [29]. The quality of clean reads was assessed using FastQC (v0.11.9) prior to alignment. Reads were aligned to the human reference genome (GRCh38) from Ensembl release 109 [30] using STAR (v2.7.10b) [31], with corresponding gene annotations from the same Ensembl release. Gene-level counts were generated during alignment by enabling the quantMode GeneCounts option in STAR. For downstream analyses, counts from the fourth column of the STAR output, corresponding to the reverse-stranded library orientation, were used.

### RNA sequencing analysis

Counts were normalized and processed using DESeq2 (version 1.40.2) [32]. Genes with an absolute log_2_ fold change of 0.58 or more as well as a false discovery rate (FDR) <0.05 were treated as significant differentially expressed genes (DEGs). DEGs were clustered using the PART algorithm [33], with parameters of recursion set to 100 and a minimum cluster size of 50 genes. The seed was set to “123456” for reproducibility purposes. Each cluster was then submitted to gprofiler (R gprofiler2 version 0.2.2) [34].

### Transcription factor analysis

The transcription factor (TF) analysis was performed using the decoupleR pipeline (version 2.14.0) [35] along with the Omnipath package (version 3.16.0) [36]. Following the tutorial provided by decoupleR, we used the run_ulm function with all parameters set to default. The top 20 TFs with positive scores and the top 20 with negative scores are retrieved. Significant DEGs associated to top five positive and top five negative TFs are plotted using a circos plots in order to compare the TFs in both groups. The circos plots were created using the circlize package (version 0.4.16) [37].

### Principal component analysis

Principal component analysis (PCA) was conducted to reduce dimensionality and identify patterns of variation in the expression data. Genes included in the PCA were mitochondrial genes meeting the DE criteria. The analysis was performed using SIMCA 18.0. The PCA scores plot visualizes sample clustering, while the loadings plot identifies key genes contributing to group differences.

### Model validation

The DModX residual plot was used to evaluate model fit by measuring residual variance. If any samples exceeded the critical limit (Dcrit), they would be flagged as potential outliers. The X/Y overview was used to assess the explanatory (R2X) and predictive (Q2X) power of the PCA model.

### Gene expression analysis via RT-qPCR

RNA was purified using the AllPrep kit (Qiagen, 80004) including DNase digestion (Qiagen, 79254) according to manufacturer’s protocol. Subsequently, reverse transcription (RT) of RNA to cDNA was carried out using high-capacity cDNA reverse transcription kit (Thermo Fisher, 4368814) according to manufacturer’s instructions. Quantitative PCR (qPCR) was performed in technical triplicates using Power SYBR PCR Master Mix (Applied Biosystems, 4368708) on a StepOnePlus v2.3 Real-Time PCR System (Applied Biosystems). Relative expression levels were determined using the 2^(−ΔΔCt)^ method. Expression of nuclear-encoded target genes was normalized to the housekeeping gene *GAPDH*, whereas expression of mitochondrial-encoded genes was normalized to *B2M*. Primer sequences are listed in Table 1. Kits and reagents are listed in Table 2.

### DNA copy number analysis

DNA was isolated from cell extracts using the AllPrep DNA/RNA Kit (Qiagen, 80004) according to the manufacturer’s instructions. Residual RNA was removed by incubation with RNase A (10 µg/ml) for 1 h at 37°C. DNA was subsequently purified by ethanol precipitation and resuspended in nuclease-free water. qPCR was performed using Power SYBR™ PCR Master Mix (Applied Biosystems) as described earlier. Relative mtDNA-CN was determined by comparing the amplification of mitochondrial gene regions (*MT-ND1* or *MT-CYB*) to a nuclear-encoded reference gene (*B2M*). Details of the primer sequences and kits are listed in Tables 1 and 2, respectively.

### RADF qPCR DNA damage assay

DNA damage in the mitochondrial genome was assessed using the RADF qPCR DNA damage assay. Genomic DNA was divided into two aliquots and incubated with or without the restriction enzyme TaqI prior to qPCR. DNA damage frequency was calculated as 2^−ΔCt^, where ΔCt = Ct(TaqI-treated) − Ct(untreated), such that reduced digestion at damaged restriction sites yields a higher damage frequency. Primer sequences are listed in Table 1.

### Mitochondrial DNA damage via long-amplicon PCR

To assess the frequency of DNA lesions capable of inhibiting polymerase activity, we employed the long-amplicon PCR assay previously described [38]. Here, the presence of DNA lesions will reduce the efficiency of PCR amplification, with the extent of amplification inversely correlating with the number of lesions within the DNA template. PCR was carried out using LongAmp Hot Start Taq2X Master Mix (New England Biolabs, M0533L). No-template controls and 50% template controls were included in each experimental run. PCR products were run on a 1% agarose gel, and band intensities were quantified using ImageJ software (version 2.14.0/1.54f). Each sample’s fluorescence value, corrected for the no-template control, was divided by its respective mtDNA-CN. The normalized values were then averaged and normalized to control sample (WT). Primer sequences are listed in Table 1.

### Whole cell extraction

Total proteins were extracted from cell pellets using RIPA lysis buffer containing 10 mM Tris–HCl, 150 mM NaCl, 0.5 mM ethylenediaminetetraacetic acid (EDTA), 0.1% sodium dodecyl sulfate (SDS), 1% Triton X-100, 1% NaDeoxycholate, 0.1 mM phenylmethylsulfonyl fluoride (PMSF), and 1× protease inhibitor cocktail (Roche). For samples where a phospho-protein was investigated, PhosSTOP (Roche, 4906845001) at a 1× concentration was used. Samples were incubated on ice for 30 min with frequent pipetting, followed by centrifugation for 10 min at 12 000 × *g* to remove cell debris. Alternatively, cell pellets were resuspended in hypotonic lysis buffer (20 mM Hepes, pH 7.9, 2 mM MgCl_2_, 0.2 mM Ethylene glycol-bis(β-aminoethyl ether)-N,N,N′,N′-tetraacetic acid (EGTA), 10% glycerol, 2 mM Dithiothreitol(DTT), 0.1 mM PMSF, 1× protease inhibitor cocktail) and incubated 5 min on ice. Samples were subjected to three freeze-thaw cycles by snap-freezing in liquid nitrogen followed by thawing in a 37°C water bath for 1 min. NaCl and NP-40 were added to final concentrations of 140 mM and 0.5%, respectively, and samples were incubated on ice for 20 min. Samples were slowly diluted with hypotonic lysis buffer + 140 mM NaCl until the final concentration of NP-40 reached 0.2%. Samples were sonicated using a Bioruptor for five pulses (30 s on, 30 s off) and centrifuged for 10 min at 12 000 × *g*. Proteins were quantified using Bradford Assay and used for further analysis.

Total protein extracts used for DNA glycosylase and trapping assays were made from cells lysed in lysis buffer (50 mM MOPS, 200 mM KCl, 0.1% Triton X-100, 1 mM EDTA, 1 mM DTT, and 1% Protease Inhibitor Cocktail). After a 30-min incubation on ice, the extracts were frozen in liquid nitrogen/thawed at 30°C three times. Cell debris was removed by 15-min centrifugation at 14 000 × g. The protein concentration was measured using Bradford assay for further analysis.

### Immunoblot analysis

Protein lysates (10–40 μg) were mixed with 4× Laemmli sample buffer to a final concentration of 1× and heated at 95°C for 5 min; for OXPHOS immunoblots, samples were instead held at 25°C to avoid heat-induced aggregation of hydrophobic membrane subunits. Equal amounts of lysate were resolved by SDS–polyacrylamide gel electrophoresis (PAGE) on NuPAGE Bis-Tris 4%–12% gradient gels (Invitrogen, NP0321) and transferred to nitrocellulose membranes using the Trans-Blot Turbo Transfer System (Bio-Rad). Membranes were blocked in 5% (w/v) non-fat dry milk in phosphate buffered saline (PBS)-T for 1 h at RT, then incubated with primary antibody for 1 h at RT or overnight at 4°C. Membranes were washed 3 × 5 min in PBS-T and incubated with HRP-conjugated secondary antibody for 30–60 min at RT. Signals were detected with SuperSignal West Femto Maximum Sensitivity Substrate (Thermo Scientific, 34094) and imaged on a Bio-Rad ChemiDoc system. For phospho-specific antibodies, 3% (w/v) bovine serum albumin (BSA) replaced milk in both blocking and antibody-dilution buffers. Band intensities were quantified in ImageJ (version 2.14.0/1.54f), and relative protein levels were normalized to tubulin as a loading control. Antibodies are listed in Table 4.

**Table 4.** List of antibodies

Antibody	Source	Identifier
**Target of interest**		
Anti-NTH1 [2660C1a]	Abcam	ab70726
**Mitochondrial**		
Anti-Total OXPHOS Western Blot Cocktail	Abcam	ab110413
Anti-Mitofilin [2E4AD5]	Abcam	ab110329
Anti-OPA1 [D-9]	Santa Cruz	sc-393296
Anti-Mitofusin 1 + 2 [3C9]	Abcam	ab57602
Anti-PGC1α	Abcam	ab191838
Anti-TFAM	ProteinTech	22586-1-AP
Anti-TFAM (used for ChIP)	Sigma–Aldrich	ABE483
Anti-Cytochrome c Apoptosis WB Antibody Cocktail	Abcam	ab110415
Anti-c-Myc [Y69]	Abcam	ab32072
Anti-POLRMT	Abcam	ab32988
**Integrated stress response**		
Anti-eIF2α	Cell Signaling Technology	9722
Anti-Phospho-eIF2α (Ser51)	Cell Signaling Technology	9721
**DNA damage**		
Anti-p-Histone H2A.X (Ser139)	Santa Cruz	sc-517348
**Epitope tags**		
Anti-Flag M2	Sigma–Aldrich	F1804
**Loading controls**		
Anti-Alpha Tubulin [DM1A], HRP	Abcam	ab40742
Anti-Histone H3 [D1H2], HRP (nuclear)	Cell Signaling Technology	12648
Anti-GAPDH (cytosolic)	Abcam	ab125247
Anti-RNA polymerase II CTD repeat YSPTSPS (phospho S5) (nuclear)	Abcam	ab5131
ChIP controls		
Rabbit IgG, monoclonal [EPR25A] - Isotype Control	Abcam	ab172730
**Secondary antibodies**		
Goat anti-Rabbit IgG (H + L), HRP	Vector Laboratories	PI-1000-1
Horse anti-Mouse IgG (H + L), HRP	Vector Laboratories	PI-2000-1
Goat anti-Mouse IgG (H+L) Cross-Adsorbed Secondary Antibody, Alexa Fluor™ 488	Invitrogen	A11001
Goat anti-Mouse IgG (H + L) highly cross-adsorbed secondary antibody, Alexa Fluor™ 488	Invitrogen	A11029

### Assessment of total OXPHOS protein abundance

Total OXPHOS protein abundance was assessed by immunoblotting using the Total OXPHOS WB Antibody Cocktail (Abcam, ab110413), which simultaneously detects representative subunits from mitochondrial respiratory chain complexes I–V (NDUFB8, SDHB, UQCRC2, MTCO1, and ATP5A). Following incubation with the OXPHOS antibody cocktail, signals corresponding to each complex were detected under identical exposure conditions across the different genotypes. For quantification, the individual band intensities of the five subunits were normalized to the loading control and expressed relative to the WT control cell line. To generate a composite OXPHOS signal for each sample, the mean value of all measured OXPHOS proteins was calculated. This composite value reflects the overall abundance of respiratory chain components rather than changes in individual proteins. Antibodies used are listed in Table 4.

### Transmission electron microscopy analysis of single mitochondrion size

Cells were fixed in 2.5% glutaraldehyde, stained in 1% osmium tetroxide, and solidified in 1% agar overnight. Agar containing the cell suspension was subjected to graded dehydration in ethanol and propylene oxide before being embedded in Durcupan resin. Ultra-thin sections were cut on a Leica RM UC6 ultramicrotome, contrasted with 1% uranylacetate and 0.3% lead citrate, and left to dry overnight. Sections were placed on an electron microscopy grid, and cellular ultrastructure was visualized on the Tecnai 12 biotwin electron microscope. For quantitative analysis, a minimum of 80 individual mitochondria per cell line were analyzed from five randomly selected fields of view. Only sections displaying intact cellular ultrastructure with clearly defined nuclear and cytoplasmic regions were included. Image analysis was performed using ImageJ (version 2.14.0/1.54f) as previously described [39]. Individual mitochondria were manually identified and outlined, and the cross-sectional area of each mitochondrion was measured.

### Bioenergetics analysis

To evaluate cellular bioenergetics, we conducted oxygen consumption rate (OCR) measurements using a Seahorse XFe96 analyzer (Seahorse Biosciences, MA, USA) via mito stress. Cells were seeded into a Seahorse microplate at a density of 7 × 10^4^ cells/well and subjected to the specified treatments. Following treatment, cells were equilibrated in DMEM lacking sodium bicarbonate, supplemented with 10 mM of D-glucose, 2 mM of glutamine, and 1 mM of sodium pyruvate. OCR measurements included basal OCR and OCR responses to sequential injections of 1.5 μM oligomycin (an ATP synthase inhibitor targeting mitochondrial complex V), 1 μM FCCP (a mitochondrial uncoupler), and 0.5 μM rotenone/antimycin (inhibitors of mitochondrial complexes I and III, respectively). Parameters such as proton leak, maximal respiration, spare respiratory capacity, non-mitochondrial oxygen consumption, and ATP-linked OCR were analyzed using the mito stress test. OCR values (pmol O_2_/min) were normalized to Hoechst 33342 levels.

### DNA glycosylase activity assay

DNA glycosylase activity was measured using standard assays as previously described [40]. In short, a 30-nt oligonucleotide containing a 5-ohU or Tg at position 15 and a 24-nt oligonucleotide containing a uracil (U) at position 14, were ^32^P-labeled at the 5′ termini using T4 polynucleotide kinase (NEB, M0201S) and [γ-^32^P] ATP (3000 Ci/mmol) (Revvity, NEG502A250UC). They were subsequently annealed with their complementary strand containing a normal base opposite the lesion. Reaction mixtures of 10 μl containing 5–10 fmol of the duplex substrate, reaction buffer (70 mM MOPS, 1 mM DTT, 1 mM EDTA, and 5% glycerol), and 10 μg total cell extracts were incubated for 2.5 h at 37°C. After addition of 10 μl of stop solution (80% formamide, 10 mM EDTA, bromophenol blue, and xylene cyanol), the reaction mixtures were incubated for 10 min at 95°C. Samples were separated on 20% denaturing polyacrylamide gels, and results analyzed using the Amersham Typhoon and the ImageQuant IQTL 8.2 software (both from Amersham Biosciences).

### NaBH_3_CN-mediated trapping of enzyme-substrate intermediates

The Schiff base covalent intermediate between the bifunctional DNA glycosylase NTHL1 and double-stranded 5-ohU containing oligonucleotide was trapped by its irreversible reduction with sodium cyanoborohydride NaBH_3_CN, as previously described [40]. Reaction mixtures containing 5–10 fmol of radiolabeled double-stranded 5-ohU substrate described earlier were incubated alone or with 10 μg total cell extracts in the presence of 6.4 mM NaBH_3_CN, 35 mM MOPS, 0.5 mM DTT, 0.5 mM EDTA, and 2.5% glycerol for 2.5 h at 37°C. Reactions were then stopped by adding 4 μl of 4× Laemmli protein sample buffer, followed by incubation for 10 min at 95°C. Samples were separated on 10% polyacrylamide gels, and results were analyzed using the Amersham Typhoon IP and the ImageQuant IQTL 8.2 software (both from Amersham Biosciences).

### Chromatin immunoprecipitation

The chromatin immunoprecipitation (ChIP) assay was performed as previously described [41] with slight modifications. Briefly, isolated mitochondria were cross-linked in a solution of 1% formaldehyde in PBS for 10 min at RT. The cross-linking reaction was stopped by adding glycine to a final concentration of 0.1 M for 5 min. Mitochondrial pellets were resuspended in SDS lysis buffer (50 mM Tris–HCl, pH 8, 10 mM EDTA, and 1% SDS) supplemented with protease inhibitors, and mtDNA sheared to 200–250 bp DNA fragments by sonication with Bioruptor sonicator (Diagenode) for 12 cycles of 30 s. Twenty micrograms of sheared mtDNA was precleared for 3 h at 4°C with Dynabeads Protein G magnetic beads (10003D, Invitrogen) and then incubated with 6 μl TFAM (Millipore-Sigma, ABE483) or IgG (Abcam, ab172730) antibodies in ChIP buffer (16.7 mM Tris–HCl, pH 8, 167 mM NaCl, 1.2 mM EDTA, 0.01% SDS, and 1.1% Triton X-100) overnight at 4°C. The DNA–protein–antibody complexes were isolated using Dynabeads Protein G magnetic beads and washed with low salt wash buffer (16.7 mM Tris–HCl, pH 8, 167 mM NaCl, 2 mM EDTA, 0.1% SDS, 1% Triton X), high salt wash buffer (16.7 mM Tris–HCl, pH 8, 500 mM NaCl, 2 mM EDTA, 0.1% SDS, 1% Triton X), and LiCl wash buffer (10 mM Tris–HCl, pH 8, 250 mM LiCl, 1 mM EDTA, 0.5% NP40, 0.5% Na-deoxycholate) for 10 min each followed by two final washes in TE buffer (10 mM Tris–HCl, pH 8, 5 mM EDTA) for 5 min each. Immunoprecipitated DNA was eluted from the beads in 300 μl elution buffer (100 mM NaHCO_3_, 1% SDS) for 20 min at RT and 10 min at 65°C. For decrosslinking, 15 μl 1 M Tris–HCl, pH 8, 15 μl 1 M NaCl, 7.5 μl of 0.5 M EDTA, and 1 μl of Proteinase K (20 mg ml^−1^) were added and samples were incubated for 1 h at 50°C and 65°C O/N. Nucleic acids were then purified with phenol/chloroform/isoamyl alcohol, precipitated by centrifugation, washed with 70% ethanol, dried and dissolved in nuclease-free water. Levels of immunoprecipitated DNA are expressed as percentage of input (% input) normalized to WT.

### MPP^+^ treatment viability assay

HEK293 cells were plated at a density of 1800 cells/well in 384-well plates and incubated overnight. Cells were subsequently treated with a dilution series of MPP^+^ (Abcam, ab144783) dissolved in medium and further incubated for 72 h. Cells were stained with final concentration 5 µg/ml Hoechst 33342 (Thermo Scientific, 62249) and 1 µM propidium iodide (Invitrogen, P3566) for 30 min and imaged. Image acquisition was performed using the ImageXpress Micro Confocal High-Content Imaging System (Molecular Devices) with a 10× CFI Plan Apochromat Lambda objective and single-band dichroic filter sets for imaging Hoechst (Ex. 377/54 nm, Em. 447/60 nm) and Propidium iodide (Ex. 560/32 nm, Em. 624/40 nm). Four fields of view were imaged per well (~72% well coverage). Image analysis was conducted with the CellProfiler^TM^ Cell Image Analysis Software 4.2.1. Cell numbers were determined as the number of nuclei stained with Hoechst, and dead cells were detected as propidium iodide positive. Cell viability was measured with the CellTiter-Glo® 2.0 Cell Viability Assay (Promega, G9243) at 25% final concentration using SPARK® Multimode Microplate Reader (Tecan) to measure luminescence. Cell seeding and MPP^+^ exposure were identical to the cell counting assay described earlier.

For HAP1 cell viability, 3 × 10^3^ cells per well were seeded into a 96-well plate and incubated for 4 h. Following this, the cells were treated with MPP^+^ at concentrations indicated in the figures, or with media alone, for 72 h. After the treatment, PrestoBlue™ Cell Viability Assay (A13262, Invitrogen) was added to each well, and the plate was incubated for an additional 2 h. Absorbance was measured at 590 nm. The results presented are averages from two distinct HAP1 clones.

### Immunofluorescence

HEK293 cells were seeded onto glass-bottomed 96-well plates (Greiner Sensoplate, Sigma–Aldrich, M4187-16EA) precoated with 40 μg/ml fibronectin (Sigma–Aldrich–Merck, F1141) overnight. For γH2AX staining, cells were treated with 10 μM Camptothecin (CPT, Sigma–Aldrich–Merck, C9911) or 220 μM MPP^+^ for 1 h at 37°C, Dimethyl sulfoxide (DMSO) served as a control. Following the treatment, cells were pre-extracted with ice-cold 0.2% Triton-X 100 for 2 min and fixed with 4% paraformaldehyde for 10 min at RT. Subsequently, cells were permeabilized using a methanol:acetone solution (1:1) for 10 min at RT and blocked with 1% BSA in PBS for 1 h at RT. Cells were stained with antibodies for 1 h, washed (3 × 5 min with PBS), and incubated with secondary antibodies for 1 h at RT. After secondary antibody incubation, cells were washed (3 × 5 min with PBS) before DNA was stained with 4′,6′-diamidino-2-phenylindole (DAPI) (Sigma–Aldrich–Merck, D9542) at a final concentration of 1 μg/ml in PBS for 5 min at RT and washed an additional four times with PBS. Antibodies used are listed in Table 4. For MitoView mitochondrial live cell analysis, cells were treated with 100 nM MitoView Green dye (Biotium) and 2 drops/ml NucBlue Live Reagent (Invitrogen) for 30 min at 37°C and immediately imaged. Images were acquired using ImageXpress Micro Confocal High-Content microscope with a 40× objective and analyzed using ImageJ software (version 2.14.0/1.54f).

**Figure 1.**
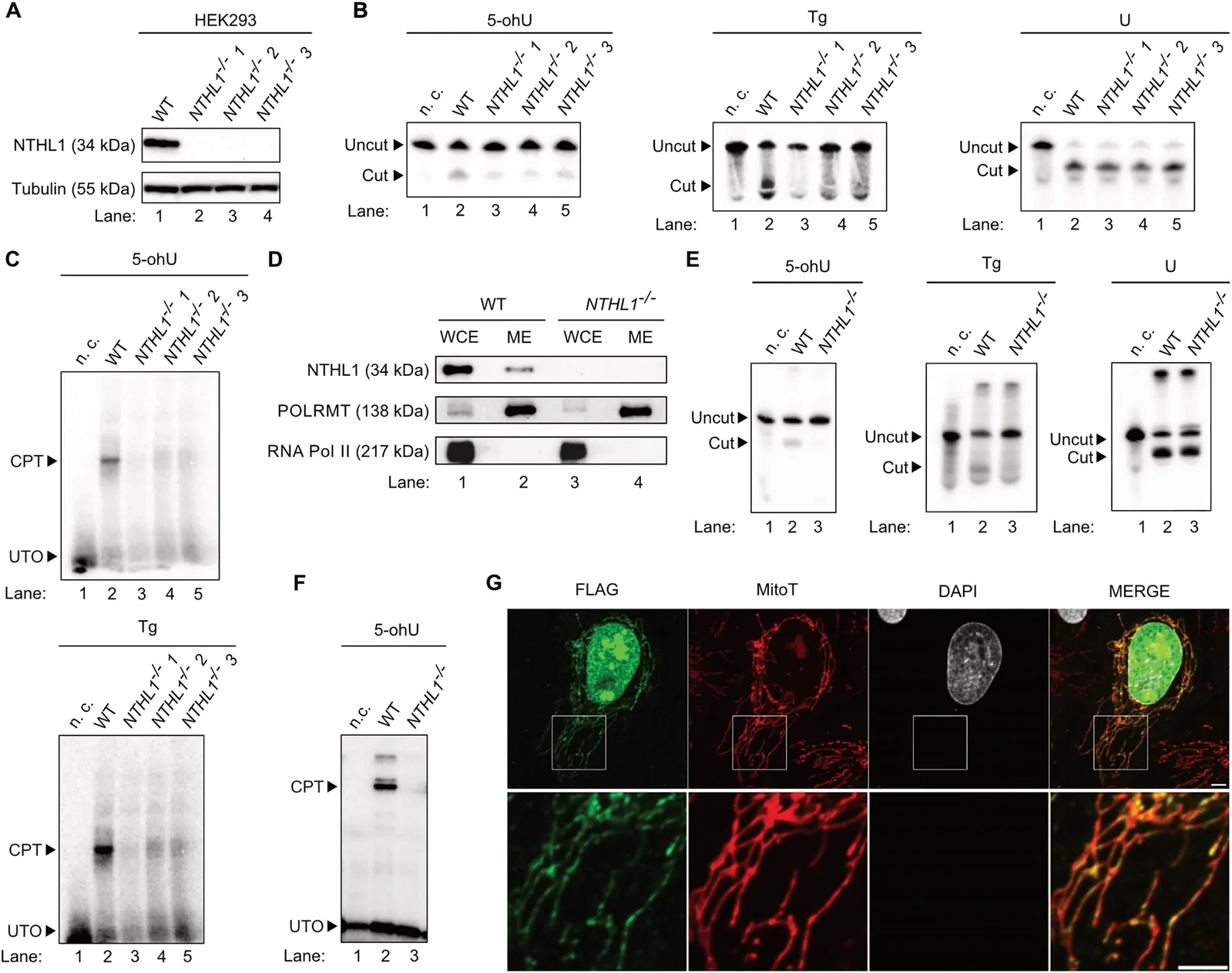
NTHL1 is active and present in mitochondria. (**A**) Immunoblot analysis confirming successful KO of NTHL1 in HEK293 cells using whole-cell extract (WCE) from WT cells (lane 1) and three independent *NTHL1^−/−^* clones (lanes 2–4). (**B**) DNA glycosylase activity assay: No-extract negative control (n.c.; lane 1) or 10 μg WCE from HEK293 WT cells (lane 2) and three independent *NTHL1^−/−^* clones (lanes 3–5) on ^32^P-end-labeled double-stranded oligonucleotides containing 5-ohU (left panel), Tg (central panel) and Uracil (U; right panel). (**C**) Detection of covalent protein–DNA intermediates by NaBH_3_CN reduction: n.c. (lane 1) or 10 μg WCE from HEK293 WT cells (lane 2) and three independent *NTHL1^−/−^* clones (lanes 3–5) on ^32^P-end-labeled double-stranded 5-ohU (left panel) and Tg (right panel). CPT, Covalent protein–DNA intermediate; UTO, untrapped oligo. (**D**) Immunoblot analysis of WCE (lanes 1, 3) or mitochondrial extracts (ME; lanes 2, 4) from HEK293 WT (lanes 1–2) and *NTHL1^−/−^* cells (lanes 3–4). POLRMT and RNA Pol II serve as mitochondrial and nuclear markers, respectively, confirming fractionation purity. (**E**) DNA glycosylase activity in mitochondrial extracts: n.c. (lane 1) or 10 μg ME from WT cells (lane 2) and *NTHL1^−/−^* cells (lane 3) on ^32^P-end-labeled double-stranded 5-ohU (Left panel), Tg (central panel) and U (right panel). (**F**) Detection of covalent protein–DNA intermediates by NaBH_3_CN reduction using n.c (lane 1), 10 μg ME from HEK293 WT (lane 2), or *NTHL1^−/−^* cells (lane 3) on ^32^P-end-labeled double-stranded 5-ohU. (**G**) IF analysis of nuclear and mitochondrial localization of NTHL1. U2OS cells were transfected with a plasmid coding for NTHL1-FLAG. 24 h after transfection, mitochondria were stained with Mitotracker Red (red), fixed and stained with an antibody against FLAG (green). Nuclei were stained with DAPI (gray). Scale bar = 5 µm.

For IF experiments related to Fig. 1G: U2OS cells were grown on four-well Ibidi µ-Slides for 1 day before transfection. Twenty-four hours after transfection, cells were incubated in 100 nM of MitoTracker Red in warm DMEM for 30 min at 37°C. Then, cells were washed with warm DMEM and immediately fixed for 20 min at 37°C with 2% formaldehyde in DMEM, rinsed twice with PBS, and permeabilized at RT in PBS 0.1% Triton X-100 for 5 min. Cells were incubated in blocking solution (PBS containing 0.1% Triton X-100, 3% BSA, and 1% normal goat serum) at 37°C for 1 h. Cells were incubated with primary antibody (anti Flag M2, Sigma–Aldrich F1804) diluted at 1/2000 in blocking solution for 1 h at 37°C, washed three times for 5 min in PBS containing 0.1% Triton, and further incubated with secondary antibody Alexa-488 goat anti-mouse (Invitrogen, A11001) diluted 1/1000 in blocking solution for 1 h at 37°C. Cells were washed three times for 5 min in PBS containing 0.1% Triton. Nuclear DNA was counterstained with 1 µg/ml DAPI. Image acquisition was performed using a NIKON Ti2/GATACA, W1 spinning disk microscope with 60× oil immersion objective (Plan Apo N.A. 1.4). Antibodies are listed in Table 4.

### Mitophagy assay and image analysis

Cells were seeded at a density of 40 000 cells per well in 96-well glass-bottom plates (microscopy grade 1.5H) pre-coated with 20 µg/ml bovine fibronectin. Mitophagy was assessed using the Mitophagy Detection Kit (Dojindo, MD01) according to the manufacturer’s instructions. This kit consists of an Mtphagy Dye for detecting mitophagy and a Lyso Dye to stain lysosomes. The Mtphagy Dye increases its red fluorescence when the mitochondria are delivered into lysosomes during mitophagy due to the acidic lysosomal environment. Co-staining with the Lyso Dye confirms the lysosomal localization of the Mtphagy signal, allowing reliable quantification of mitochondria that have undergone fusion with lysosomes as part of mitophagy.

Briefly, culture medium was removed, and cells were washed with Opti-MEM prior to incubation in Opti-MEM containing 100 nM Mtphagy dye (Mitophagy staining) for 30 min at 37°C in a humidified incubator with 5% CO_2_. Following staining, cells were washed twice with Opti-MEM. Cells were then incubated in Opti-MEM supplemented with Hoechst 33 342 and Lyso dye (lysosomal staining) at a final concentration of 1 µM. Cells were incubated for 1 h and subsequently subjected to live-cell imaging for 60 min.

### Cell proliferation assay

HEK293 WT and *NTHL1^−/−^* cells were seeded at 200 000 cells per well of a six-well plate. At 24, 48, and 72 h post-seeding, cells were counted using the Thermo Fisher Countess 3. Briefly, an equal volume of 0.4% trypan blue was added to cell suspension, gently mixed, and loaded on a Countess chamber slide to determine the number of viable cells.

### N-acetyl cysteine and XJB-5-131 treatment

HEK293 cells were seeded at 200 000 cells per well of a six-well plate and incubated for 24 h before treatment with freshly prepared N-acetyl cysteine (NAC) at concentrations of 1, 5, and 10 mM or XJB-5-131 at concentrations of 0.1, 0.5, and 1 μM for 3 and 6 h. Cells were collected and subject to either DNA extraction followed by qPCR or whole cell extraction and immunoblot analysis.

### Statistical analysis

Unless otherwise stated, all the experiments presented are representative of *n* ≥ 3 independent experiments. Data analysis was performed using GraphPad Prism 10.2.2. (GraphPad Software, Inc., La Jolla, CA). Statistical significance was determined by one-way analysis of variance (ANOVA) with Dunnett’s multiple comparison test (Figs 3–7 and Supplementary Figs S1–S5) and *t*-test (Supplementary Figs S3C and S6). Each dot represents an independent biological replicate in all panels, except in Fig. 4D, where each dot corresponds to a single ROI, and in Fig. 4F, where each dot represents an individual mitochondrion. Data are presented as mean ± SD, except for Fig. 5A and B and Supplementary Fig. S4B and C, which show mean ± SEM. *P ≤ .05; **P ≤ .01; ***P ≤ .001; ^****^P ≤ .0001; ns—not significant.

## Results

### NTHL1 is active and present in mitochondria

We employed CRISPR**-**Cas9 to generate *NTHL1^−/−^* cell lines (Fig. 1A), targeting all three *NTHL1* mRNA isoforms (Supplementary Fig. S1A). Following transfection of HEK293 WT cells with *NTHL1*-specific sgRNA and Cas9 nuclease, individual clones were isolated and initially screened using an indel-specific PCR (Supplementary Fig. S1B). Clones lacking amplification in the indel-specific PCR were considered potential KOs (Supplementary Fig. S1C) and were further validated by Sanger sequencing. Biallelic indel mutations at the CRISPR target site were characterized using DECODR [27] (Supplementary Fig. S1D). Immunoblotting verified loss of NTHL1 expression in KO clones (Fig. 1A). To ensure specificity, potential off-target effects were predicted using CRISPOR. Top off-target candidates within exon regions were ruled out via indel-specific PCR and Sanger sequencing of the amplified off-target sites (Supplementary Fig. S1E–H). Of note, our approach ensured the KO of all three isoforms to avoid any potential compensatory regulation. This was important because, despite isoform 1 being the most highly expressed, the other isoforms were still present and detectable (Supplementary Fig. S1I–L). To interrogate the consequences of NTHL1 KO, DNA glycosylase activity and the formation of covalent protein–DNA intermediates were evaluated. A significant reduction in DNA glycosylase activity was observed in *NTHL1^−/−^* cells specifically for oligonucleotides containing 5-ohU and Tg, whereas activity against uracil (U)-containing substrates remained comparable to WT levels (Fig. 1B). Additionally, trapping assay showed selective trapping of 5-ohU- and Tg-containing DNA oligonucleotides in WT cell extracts, whereas this trapping was dramatically reduced in KO cell extracts (Fig. 1C). Although we cannot exclude the possibility that other DNA glycosylases may contribute to the residual signal observed, the substantial reduction in the activity assay and the complete loss in the trapping assay observed in the *NTHL1^−/−^* cells, confirm the functional loss of NTHL1.

To verify the mitochondrial localization and activity of NTHL1 protein, we isolated mitochondria from WT cells. Immunoblotting revealed that a small fraction of NTHL1 localizes to the mitochondria (Fig. 1D). To ensure signal specificity, the immunoblot was performed in NTHL1-deficient cell lines, where no detectable signal was observed (Fig. 1D). To assess enzymatic activity, DNA glycosylase and trapping assays were conducted using purified mitochondrial extracts from both WT and *NTHL1^−/−^* cell lines (Fig. 1E and F). These assays confirmed NTHL1 enzymatic activity exclusively in WT mitochondrial extracts, validating mitochondrial isolation and functional presence of NTHL1 in mitochondria. To further confirm the mitochondrial localization of NTHL1, IF analysis was performed, and a clear colocalization of NTHL1 with mitochondrial markers was observed (Fig. 1G).

### RNA sequencing analysis reveals altered mitochondrial gene expression in *NTHL1^−/−^* cell lines

**Figure 2.**
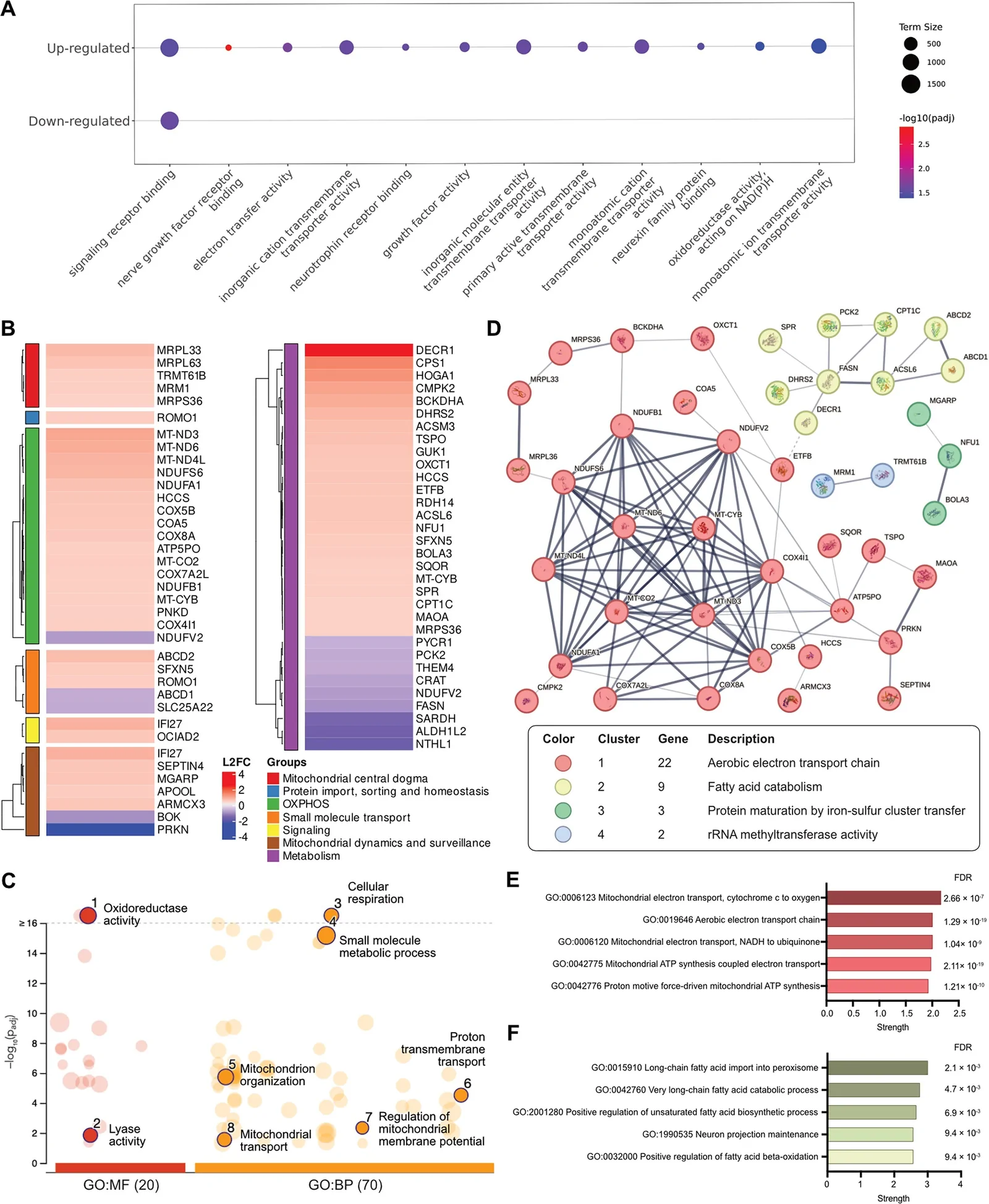
Impact of NTHL1 KO on the expression of mitochondrial-related genes. (**A**) Pathway enrichment bubble plot of GO molecular function terms for genes upregulated and downregulated in *NTHL1^−/−^* cells [log_2_ fold-change threshold ± 0.58, FDR < 0.05; (*n* = 4 replicates per cell line)]. Bubble color represents adjusted *P-*values, and bubble size corresponds to gene counts. (**B**) Heatmap of DEGs involved in mitochondrial function presented as the average log_2_ fold-change of *NTHL1^−/−^* cell lines (*n* = 4 replicates per cell line). Red indicates significantly increased mRNA expression, while blue indicates significantly decreased mRNA expression in *NTHL1^−/−^* cells compared with WT controls (log_2_ fold-change threshold ± 0.5, FDR < 0.05). The color scale legend is shown in the bottom right. (**C**) GO enrichment analysis: The *x*-axis represents the GO term (molecular function and biological process), and the *y*-axis represents adjusted *P*-value (-log_10_ Padj). Top significant driver terms are highlighted (adjusted *P*-value < .05). (D–F) Network analysis and GO analysis of mitochondrial-related genes altered in *NTHL1^−/−^* cells. (**D**) STRING network analysis of mitochondrial DEGs in *NTHL1^−/−^* cells. The network is subdivided in four clusters (depicted in red, light green, blue, and dark green) through k-means clustering, with the members that characterize each cluster presented. Edge thickness is representative of interaction confidence. (**E**) GO enrichment analysis for biological processes (represented with strength as a scale) shows significant enrichment for terms related to aerobic ETC and oxidoreductase activity in Cluster 1. (**F**) GO analysis of biological processes related to Cluster 2 highlights significant enrichment for processes involved in fatty acid metabolism, lipid catabolism, and arachidonate-CoA ligase activity, with strength represented on a scale. The analysis was performed using two independent *NTHL1^−/−^* clones.

To identify whether loss of NTHL1 affects transcriptional levels of genes involved in mitochondrial dynamics, we performed RNA sequencing on WT and *NTHL1*^−/−^ cell lines. DEGs were identified by comparative transcriptomics using a log_2_ fold-change (log_2_ FC) threshold of ± 0.58, corresponding to a minimum 1.5-fold change in either direction in messenger RNA (mRNA) levels, and a FDR < 0.05. Gene Ontology (GO) molecular function enrichment analysis revealed that the DEGs were predominantly upregulated and significantly enriched for mitochondrial-related functions including electron transfer activity, oxidoreductase activity relating to NAD(P)H, and transmembrane transport of monoatomic cations and inorganic molecules (Fig. 2A and Supplementary Table S1). These processes are hallmarks of mitochondrial bioenergetics and ion homeostasis. Specifically, electron transfer and oxidoreductase activities are central to the mitochondrial respiratory chain, supporting ATP production and maintaining redox balance, while transmembrane cation transporters regulate mitochondrial membrane potential and ionic gradients essential for metabolic and apoptotic signaling. Moreover, we observed an enrichment of neurotrophin and nerve growth factor receptor binding among the upregulated genes. To further characterize the broad impact on the transcript levels of genes involved in mitochondrial dynamics, DEGs (log_2_ FC ± 0.5, FDR < 0.05) were filtered using MitoCarta 3.0. Sixty-eight mitochondrial-related genes were found to be differentially expressed in *NTHL1^−/−^* cell lines compared to WT (Fig. 2B). Subsequent GO enrichment analysis performed using g:Profiler [42] indicated that most DEGs in *NTHL1^−/−^* cell lines were associated with oxidoreductase activity as a molecular function (adjusted *P*-value 6.329 × 10^–19^) (Fig. 2C). The DEGs were also enriched for biological processes such as the small molecule metabolic process (adjusted *P*-value 5.995 × 10^–21^) (Fig. 2C and Supplementary Fig. S2A and B). These findings indicate a selective reprogramming of mitochondrial-related transcriptional pathways in *NTHL1^−/−^* cells, rather than a global upregulation of mitochondrial gene expression.

To investigate the impact of NTHL1 deficiency on transcript levels of mitochondrial-related genes, we performed principal component analysis (PCA) on mitochondrial DEGs in *NTHL1^−/−^* and WT samples. The PCA scores plot (Supplementary Fig. S2C) revealed a clear separation between KO and WT samples along the first predictive component, indicating distinct mitochondrial gene expression profiles between the groups. The clustering of biological replicates within each condition highlights the consistency of transcriptional changes in response to NTHL1 deficiency. The corresponding loadings plot (Supplementary Fig. S2D) identified genes contributing to the observed separation. Genes associated with KO samples, such as *ND6, ND3*, and *ND4L*, are involved in mitochondrial ETC function, reflecting mitochondrial respiratory activity. Genes associated with WT samples, including *FASN* and *ALDH1L2*, represent metabolic functions such as lipid biosynthesis and one-carbon metabolism. These findings suggest that NTHL1 deficiency alters mitochondrial gene expression, potentially leading to functional reorganization of mitochondrial processes. We used DModX residual analysis to evaluate potential outliers, and all samples falling below the critical threshold (DCrit = 0.05), confirming the robustness of the analysis (Supplementary Fig. S2E). The RNA sequencing results were confirmed via reverse transcription qPCR (RT-qPCR) of genes belonging to the top identified biological processes, specifically those showing increases in mRNA levels for mitochondrial-encoded genes *MT-ND6* and *MT-CYB* (Supplementary Fig. S3A and B) and nuclear-encoded gene*s COX4I1, COX5B, NDUFS6, HCCS, APOOL, NFU1, MAOA, ACSM3, ACSL6, HMOX1, TSPO*, and *OXCT1* (Supplementary Fig. S3C–N), as well as nuclear-encoded gene *FASN* showing decreased mRNA levels (Supplementary Fig. S3O).

To further explore the functional relationships among the mitochondrial-related genes affected by NTHL1 loss, we performed a STRING network analysis to identify key interaction networks among the differentially expressed mitochondrial-related genes (Fig. 2D). Four distinct clusters emerged, with cluster 1 (Aerobic electron transport chain) being the largest, comprising 22 genes and primarily associated with mitochondrial functions crucial for energy production (Fig. 2E). This cluster was significantly enriched for genes involved in the aerobic ETC and oxidoreduction-driven active transmembrane transporter activity, both critical for mitochondrial respiration and ATP synthesis. Cluster 2 (gene count: 9) included genes involved in fatty acid catabolism and arachidonate-CoA ligase activity, which are essential for mitochondrial lipid metabolism (Fig. 2F). In addition to these main clusters, two smaller clusters were identified: Cluster 3 (gene count: 3) was associated with protein maturation by iron-sulfur cluster transfer, and Cluster 4 (gene count: 2) was linked to rRNA methyltransferase activity, a process crucial for mitochondrial protein synthesis. Alterations in this cluster point to potential defects in mitochondrial translation and protein maturation in *NTHL1^−/−^* cells. Hence, NTHL1 deficiency affects multiple mitochondrial processes, including energy production and fatty acid metabolism.

### NTHL1 deficiency causes increased mtDNA-CN and mitochondrial biogenesis- and fusion-associated proteins

**Figure 3.**
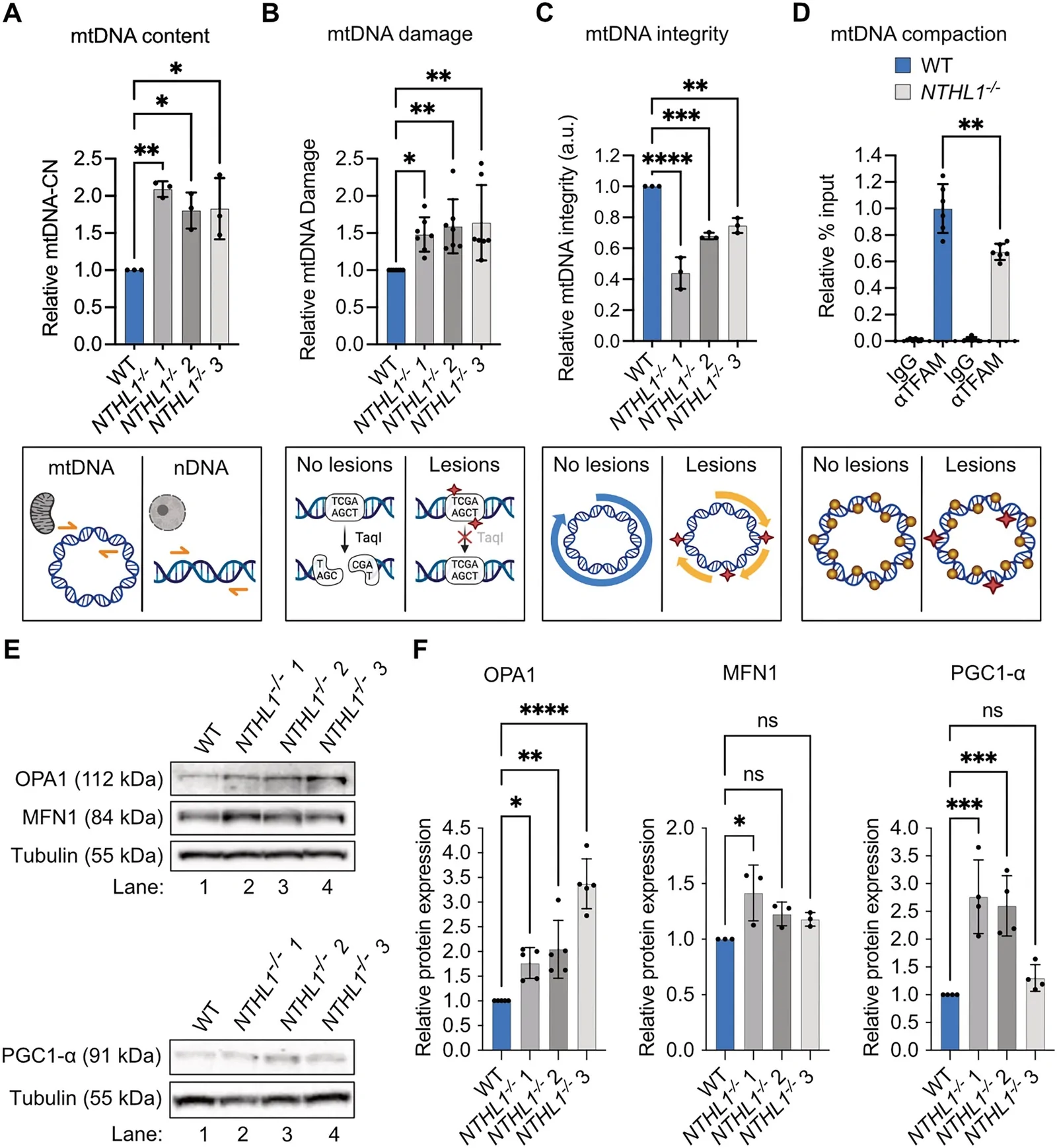
*NTHL1^−/−^* cells show increased mitochondrial biogenesis. (**A**) Relative mtDNA-CN of WT and three independent *NTHL1^−/−^* clones (*n* = 3). (**B**) Relative mtDNA damage measured with the RADF assay of WT and three independent *NTHL1^−/−^* clones (*n* = 7). (**C**) Relative mtDNA integrity measured via long-amplicon PCR in WT and three independent *NTHL1^−/−^* clones (*n* = 3). (**D**) ChIP on mtDNA for TFAM in WT and *NTHL1^−/−^* cells (*n* = 5). (A–D) Below each bar graph is a graphical representation showing the method used to perform the assay. (**E**) Immunoblot analysis of OPA1, MFN1, and PGC1α protein levels in WT and *NTHL1^−/−^* cell extracts (OPA1, *n* = 5; MFN1, *n* = 3; PGC1α, *n* = 3). (**F**) Relative quantification of data depicted in panel (E). Each dot represents an independent biological replicate. Error bars represent mean ± SD (*n* ≥ 3). *P ≤ .05; **P ≤ .01; ***P ≤ .001; ^****^P ≤ .0001, one-way ANOVA.

Among the DEGs, several mitochondrially encoded genes displayed increased levels. Given that mitochondrial gene expression is tightly linked to mitochondrial function, we reasoned that this overall increase in mRNA levels might be a compensatory response to mitochondrial dysfunction, potentially driven by an increase in mitochondrial biogenesis or a response to elevated oxidative stress. Changes in mitochondrial biogenesis are commonly reflected by alterations in mtDNA-CN, which has long been recognized as a biomarker for mitochondrial dysfunction [18, 43, 44]. Therefore, we assessed mtDNA-CN, which revealed an average of 1.9-fold increase in *NTHL1^−/−^* cells compared to WT cell lines (Fig. 3A). To assess mtDNA integrity, we used two PCR-based methods to quantify DNA damage. First, the real time qPCR Analysis of Damage Frequency (RADF) assay [45], which measures DNA lesions that block TaqI enzyme digestion at 5′-TCGA-3′ sites, revealed increased mtDNA damage in NTHL1-deficient cells (Fig. 3B). Similarly, long-amp PCR, which detects DNA damage by measuring reduced amplification of long mtDNA fragments, showed significantly lower amplification in *NTHL1^−/−^* cells compared to WT (Fig. 3C). This reduction reflects impaired polymerase processivity due to DNA damage, indicating an increased proportion of lesions per mtDNA molecule. Together, these assays consistently indicate elevated mtDNA damage upon loss of NTHL1. Importantly, this effect was not caused by compromised mitophagy or differences in cell proliferation (Supplementary Fig. S4A–D).

To evaluate whether DNA lesions affect mtDNA compaction, we performed ChIP targeting TFAM, a mtDNA coating and compacting protein [46, 47]. Our analysis revealed a significant reduction in TFAM binding in NTHL1^−/−^ cell lines compared to WT cells (Fig. 3D). This decrease was not attributed to altered TFAM expression levels, as RT-qPCR and immunoblotting analyses showed no changes in expression levels between WT and *NTHL1^−/−^* cell lines (Supplementary Fig. S4E–G).

Next, to determine whether mitochondrial dynamics are further affected, we performed immunoblotting for mitochondrial fusion proteins. We observed an average two-fold increase in the protein levels of Dynamin-like GTPase OPA1 (Fig. 3E and F), a mitochondrial inner membrane protein that plays a central role in inner membrane fusion and cristae morphology [48–52]. Notably, loss of OPA1 disrupts respiratory chain assembly, leading to reduced ETC activity and impaired OXPHOS [53]. Furthermore, fibroblasts from patients carrying OPA1 mutations have previously been shown to have decreased mtDNA levels [54]. Additionally, our results showed no significant differences in the protein levels of Mitofusin 1 (MFN1), a key mitochondrial outer membrane protein that mediates mitochondrial fusion, between *NTHL1^−/−^* and WT cells, except for one clone (Fig. 3E and F). We also observed an average two-fold increase in the protein levels of peroxisome proliferator-activated receptor γ coactivator 1α (PGC1α), a key regulator of mitochondrial biogenesis (Fig. 3E and F). Under oxidative stress, PGC1α drives the expression of numerous nuclear-encoded mitochondrial genes, including those involved in mitochondrial biogenesis and OXPHOS, aligning with our transcriptomics results. Taken together with the increase in mtDNA-CN, our results indicate that NTHL1 plays a role in regulating mitochondrial biogenesis, potentially mediated by PGC1α. Additionally, the increase in OPA1 levels suggests that loss of NTHL1 might promote mitochondrial fusion.

### Loss of NTHL1 is associated with increased mitochondrial mass and respiration

**Figure 4.**
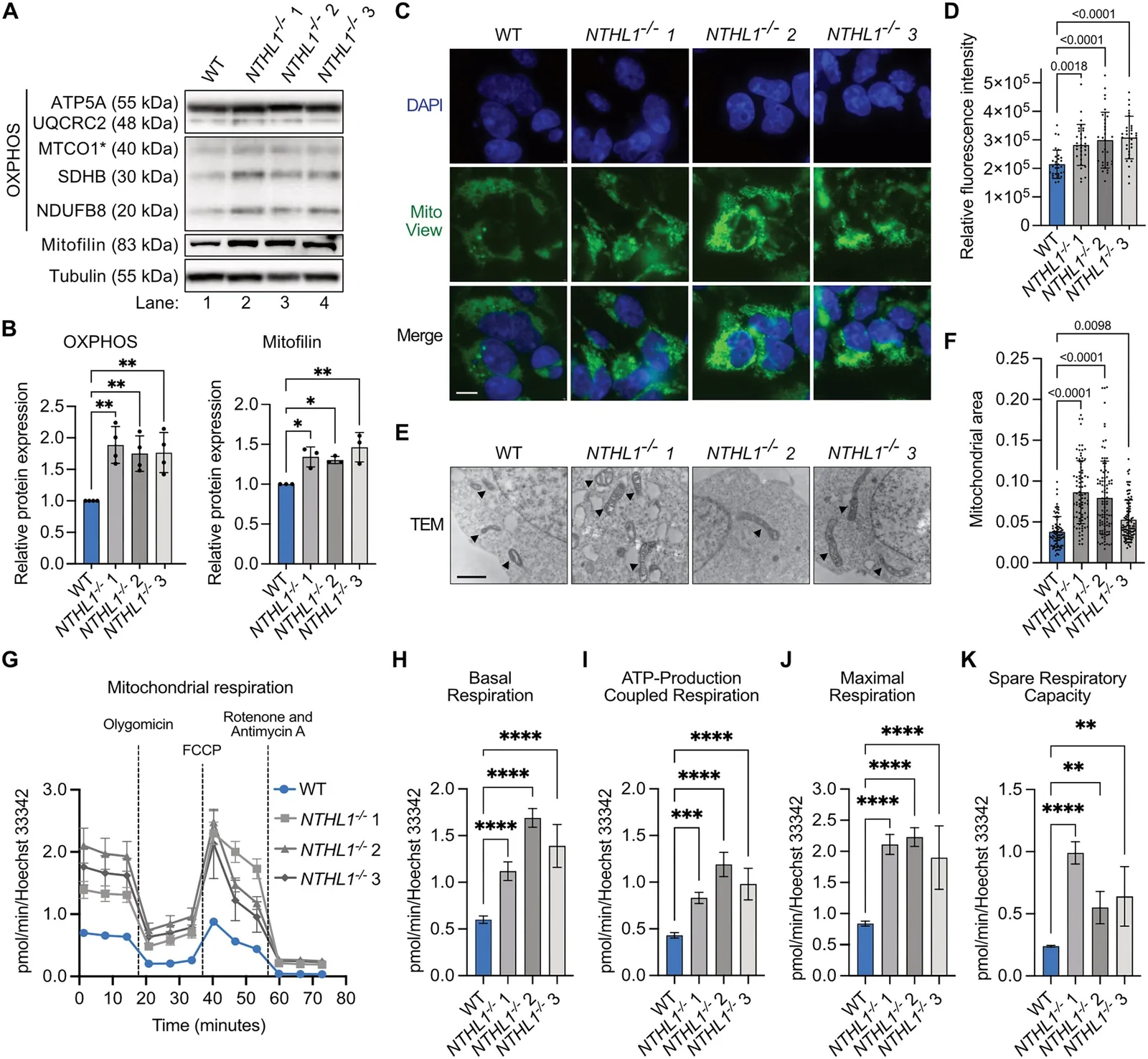
Mitochondrial mass and energy metabolism are enhanced in *NTHL1^−/−^* cells. (**A**) Representative immunoblots of OXPHOS complex subunits and Mitofilin in WT (lane 1) and *NTHL1^−/−^* clones (lanes 2–4). Subunits probed: complex I (CI, NDUFB8), complex II (CII, SDHB), complex III (CIII, UQCRC2), complex IV (CIV, MTCO1*), and complex V (CV, ATP5A). *MTCO1 is mitochondrially encoded. Tubulin served as a loading control. Blots originate from independent runs. (**B**) Quantification of total OXPHOS subunit and Mitofilin protein levels from immunoblots in panel (A) (OXPHOS, *n* = 4; Mitofilin, *n* = 3). Each dot represents an independent biological replicate. (**C**) Representative IF images of WT and *NTHL1^−/−^* clones stained with MitoView (green) to label mitochondria and DAPI (blue) to visualize nuclei. Merged images show increased mitochondria signal intensity in *NTHL1^−/−^* lines. Scale bar = 10 µm. (**D**) Quantification of mitochondrial mass from the experiment shown in panel (C). Each dot represents a single ROI. (**E**) Representative TEM images of WT and *NTHL1^−/−^* cells. Arrows indicate single mitochondria. Scale bar = 2 μm. (**F**) Quantification of mitochondrial size from the TEM images in panel (E). Each dot represents individual mitochondria. (G–K) Respirometry under mitochondrial stress (*n* = 5). (**G**) Respirometry traces representing oxygen consumption curves. The first three measurement points represent basal mitochondrial respiration. The following three points, after the addition of oligomycin to inhibit ATP synthase, represent oligomycin-insensitive oxygen consumption. The difference between basal respiration and oligomycin-insensitive respiration represents ATP production–coupled respiration. The next three points represent maximal mitochondrial respiratory capacity, achieved by uncoupling the mitochondrial inner membrane with FCCP. The final three points measure non-mitochondrial oxygen consumption when the mitochondrial respiratory chain is inhibited by rotenone and antimycin A. Quantitative analyses of basal respiration (**H**), ATP-production coupled respiration (**I**), maximal respiration (**J**), and spare respiratory capacity (**K**). Error bars represent mean ± SD (*n* ≥ 3). *P ≤ .05; **P ≤ .01; ***P ≤ .001; ^****^P ≤ .0001, one-way ANOVA.

To further investigate the role of NTHL1 in regulating mitochondrial function, we first assessed the abundance of OXPHOS complex subunits by immunoblotting WT and *NTHL1^−/−^* clones with an OXPHOS antibody cocktail spanning complexes I–V. This analysis revealed a consistent global increase in OXPHOS subunit levels in NTHL1-deficient cells, which was paralleled by elevated levels of the mitochondrial inner-membrane protein Mitofilin, together indicative of increased mitochondrial content (Fig. 4A and B). Consistent with this, IF staining with MitoView Green showed a marked increase in mitochondrial signal in *NTHL1^−/−^* cell lines (Fig. 4C and D). TEM further revealed changes in mitochondrial morphology, with *NTHL1^−/−^* cells showing an increased mitochondrial area (Fig. 4E and F).

To examine the impact of increased mitochondrial mass in *NTHL1^−/−^* cells, we assessed mitochondrial metabolic activity using the Seahorse XF Cell Mito Stress Assay (Fig. 4G–K). The *NTHL1^−/−^* cell lines showed, on average 80% higher basal mitochondrial OCR compared to WT cells (Fig. 4G and H). This positive trend was also evident for mitochondrial OCR sensitive to the ATPase inhibitor oligomycin, where the OCR coupled to ATP production was significantly elevated in *NTHL1^−/−^* cells compared to WT cells (see OCR after addition of oligomycin in Fig. 4G, and bars in Fig. 4I). Moreover, when mitochondria were stressed by permeabilizing the inner membrane for H^+^ with carbonyl cyanide 4-(trifluoromethoxy) phenylhydrazone (FCCP) to reveal maximal mitochondrial respiratory capacity, the increase in OCR of *NTHL1*^−/−^ mitochondria was on average 2 times higher compared to WT cells (see OCR after addition of FCCP in Fig. 4G and bars in Fig. 4J). Additionally, the spare respiratory capacity was significantly increased in *NTHL1^−/−^* cells (see maximal OCR after FCCP minus basal in Fig. 4G and bars in Fig. 4K). These findings suggest that the absence of NTHL1 enhances mitochondrial respiratory activity and overall mitochondrial efficiency. This enhancement suggests that while NTHL1 is essential for maintaining mtDNA integrity, its absence triggers adaptive mechanisms to sustain mitochondrial function and energy production. Thus, the phenotype could be a compensatory response to increased mtDNA damage observed in *NTHL1^−/−^* cells.

Furthermore, the Seahorse analysis revealed that *NTHL1^−/−^* cells showed increased proton leak and non-mitochondrial oxygen consumption (Supplementary Fig. S5A and B). Increased proton leak suggests that more protons are crossing the mitochondrial membrane independently of ATP production, resulting in a “leak” of energy as heat, bypassing the ATP synthase. Non-mitochondrial oxygen consumption reflects the oxygen consumed by reactions outside the mitochondria. These reactions could include oxidase activities, such as NADPH oxidases or other enzymes that generate ROS, indicating heightened oxidative stress.

Taken together, these observations indicate that the cells are experiencing mild oxidative stress, which may be driving adaptive changes in cellular metabolism and energy production. This shift is reminiscent of mitohormesis: a compensatory response where moderate mitochondrial stress enhances cellular resilience and function. This interpretation aligns with previous observations showing that NTH-1 loss enhances resilience in a *C. elegans* model [10].

### Loss of NTHL1 reduces sensitivity to complex I inhibition

**Figure 5.**
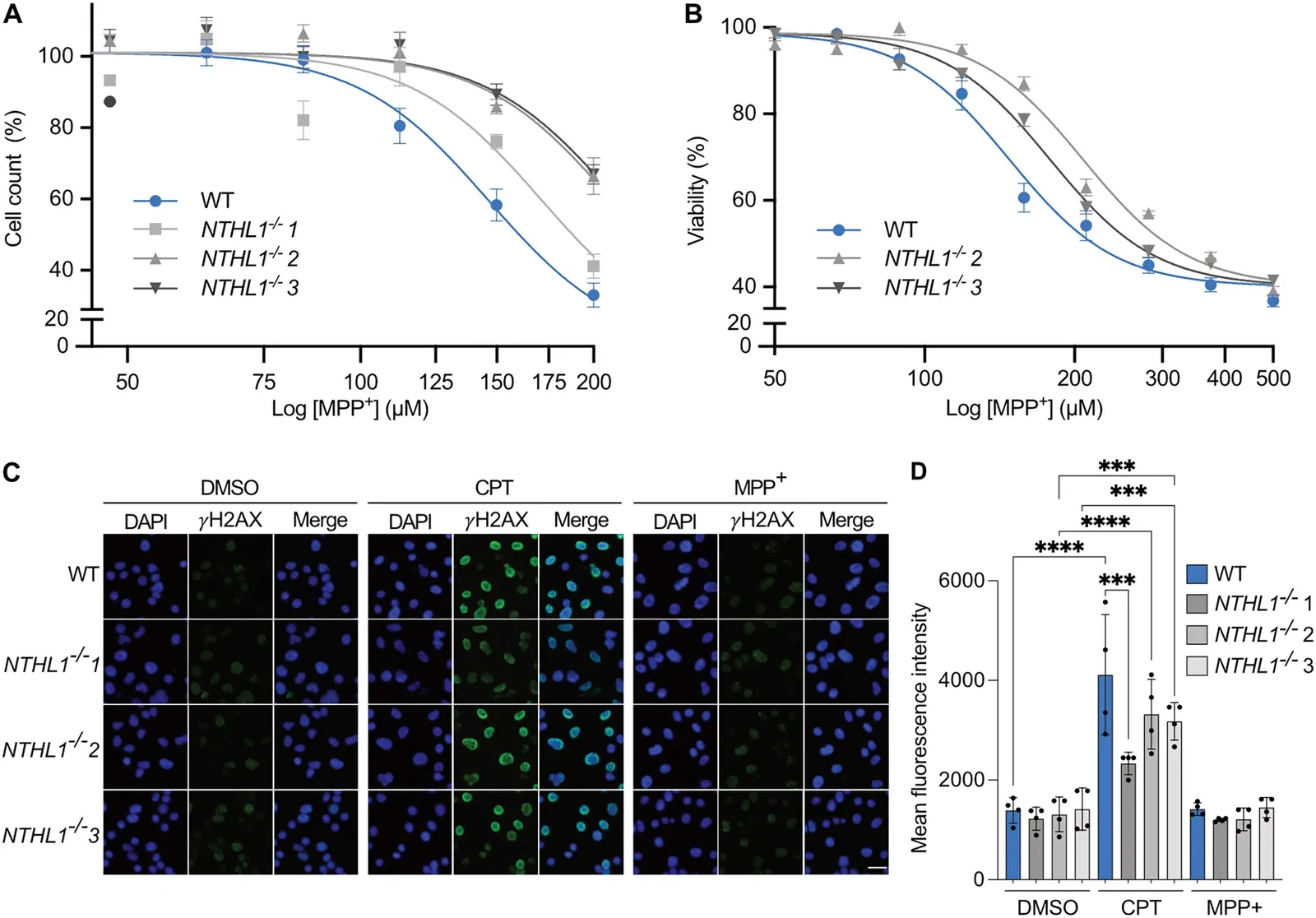
NTHL1^−/−^ cells display enhanced tolerance to MPP^+^. (**A**) WT and three independent *NTHL1^−/−^* clones were treated with the indicated concentrations of MPP⁺ for 72 h, stained with Hoechst 33342, and the number of cells per well was counted. MPP⁺ treatment reduced cell numbers in WT cells, whereas NTHL1^−/− ^cells were affected only at the highest concentrations tested.(**B**) WT and *NTHL1^−/−^* cells were exposed to the indicated concentrations of MPP^+^ for 72 h, and viability was assessed with Cell-titer glo. Viability decreased in both genotypes, but *NTHL1*^−/−^ cells remained significantly more viable than WT at all tested concentrations.(**C**) Representative IF image of γH2AX in WT and *NTHL1^−/−^* cells following CPT or MPP^+^ treatment. (**D**) Quantification of the analysis depicted in panel (C). Each dot represents an independent biological replicate. Error bars represent mean ± SEM (*n* = 2) (A, B) or ± SD (*n* = 4) (**D**). ***P ≤ .001; ^****^P ≤ .0001, one-way ANOVA.

If the changes observed were indeed part of a mitohormetic response, we would expect that the *NTHL1^−/−^* cells would exhibit altered response to mitochondrial stress due to the observed increase in OXPHOS-related proteins in these cells. MPP^+^ is a mitochondrial toxin known to inhibit mitochondrial complex I (NADH oxidoreductase) of the ETC [55–57]. MPP^+^ disrupts electron flow by inhibiting complex I, reducing ATP production and increasing ROS, leading to oxidative stress and cellular damage. We hypothesized that NTHL1 deficiency might alter the cellular response to MPP^+^-induced mitochondrial dysfunction. Consistent with our hypothesis, NTHL1-deficient cells exhibited enhanced tolerance to MPP^+^ compared to WT cells. After 72 h of MPP^+^ treatment, WT cells showed a significant reduction in cell numbers, while *NTHL1^−/−^* cells showed markedly reduced sensitivity across various MPP^+^ concentrations (Fig. 5A). Furthermore, cell viability was decreased in both WT and *NTHL1^−/−^* cells following MPP^+^ treatment, but *NTHL1^−/−^* cells maintained significantly higher viability than WT cells at all tested concentrations (Fig. 5B). Similar resistance was observed in HAP1 cell lines (Supplementary Fig. S6A–C).

We reasoned that this effect was primarily mitochondrial and that MPP^+^ did not trigger a nuclear DNA damage response. To confirm this, we treated cells with MPP^+^ and performed IF staining for γH2AX, a marker of DNA damage. No induction of γH2AX foci was observed in WT or *NTHL1^−/−^* cells upon MPP^+^ treatment (Fig. 5C and D). As a control, we treated cells with CPT, a topoisomerase I inhibitor that induces DNA damage by stabilizing the cleavage complex between topoisomerase I and DNA, leading to replication-associated double-strand breaks. As expected, CPT treatment led to a significant increase in γH2AX foci (Fig. 5C and D). Together, these results indicate that NTHL1 loss enhances cellular resilience to mitochondrial complex I inhibition, without eliciting a nuclear DNA damage response, suggesting a mitohormetic mechanism that promotes survival under mitochondrial stress.

### NTHL1 deficiency-induced mitohormesis is rescued by mitochondrial ROS scavengers and reintroduction of mitochondrial NTHL1

**Figure 6.**
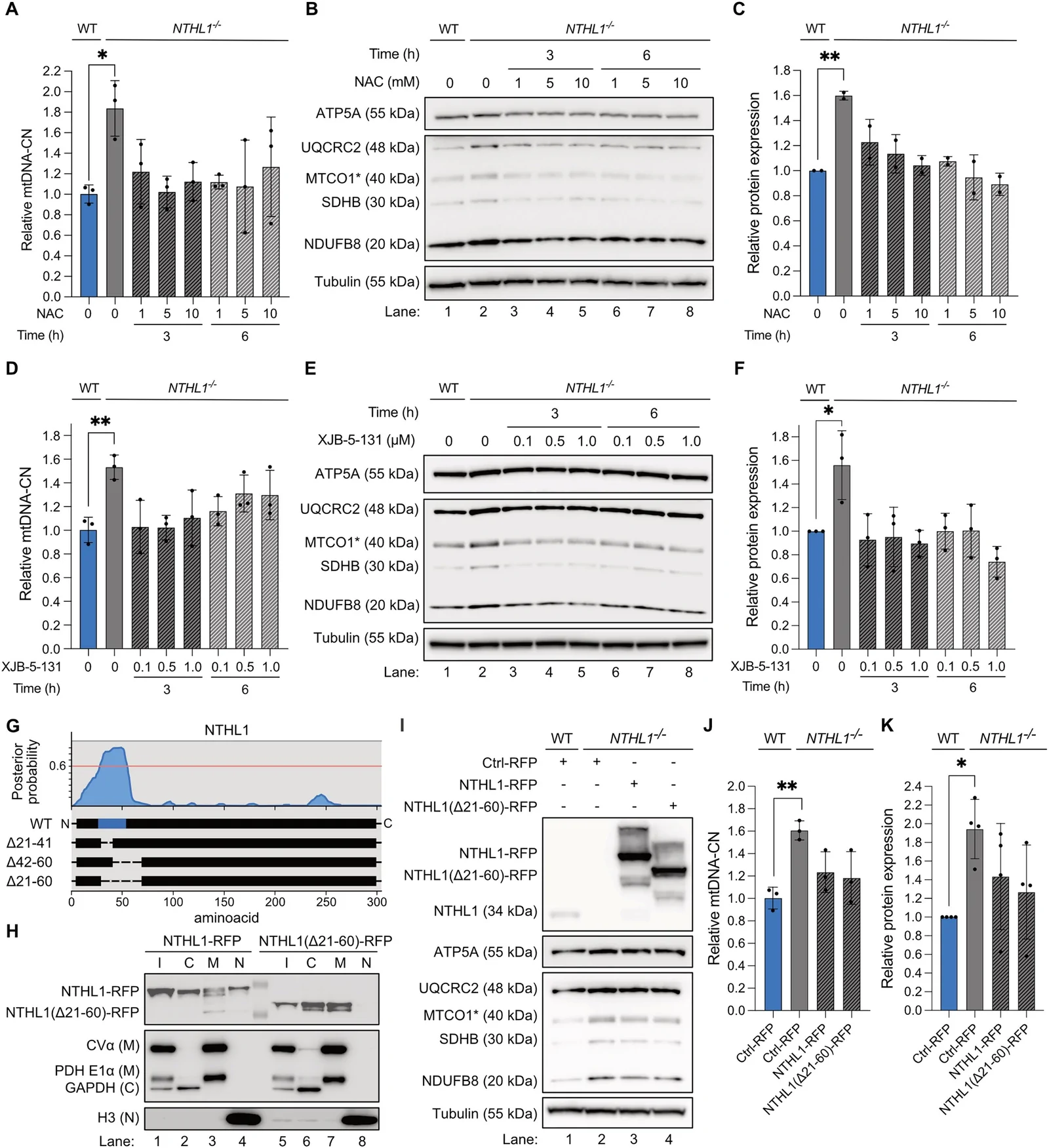
NTHL1 deficiency-induced mitohormesis is rescued by ROS scavengers and mitochondrial isoform reintroduction. (**A**) Analysis of mitochondrial mtDNA-CN in WT and *NTHL1^−/−^* cells following treatment with ROS scavenger NAC at the indicated concentrations (1, 5, and 10 mM) and time points (3 and 6 h) (*n* = 3). (**B**) Representative immunoblots of OXPHOS protein subunits in WT and *NTHL1^−/−^* cells treated with NAC. Tubulin serves as the loading control. (**C**) Quantification of relative protein expression from the western blots shown in panel (B) (*n* = 2). Data are normalized to WT controls. (**D**) Analysis of mitochondrial mtDNA-CN in WT and *NTHL1^−/−^* cells following treatment with the mitochondrial ROS scavenger XJB-5-131 at the indicated concentrations (0.1, 0.5, and 1.0 μM) and time points (3 and 6 h) (*n* = 3). (**E**) Representative immunoblots of OXPHOS protein subunits in WT and *NTHL1^−/−^* cells treated with XJB-5-131. Tubulin serves as the loading control. (**F**) Quantification of relative protein expression from the western blots shown in panel (E) (*n* = 3). Data are normalized to WT controls. (**G**) Schematic overview of the NTHL1 protein highlighting the predicted NLS regions, previously characterized truncation mutants Δ21–41 and Δ42–60, and the newly generated Δ21–60 deletion construct. (**H**) Representative immunoblots of fractionated *NTHL1^−/−^* cell lysates (I: Input, C: Cytosol, M: Mitochondria, N: Nucleus) expressing NTHL1-RFP or the NTHL1(Δ21–60)-RFP deletion mutant. Cellular fractionation was validated using ATP synthase subunit alpha (CVα) and pyruvate dehydrogenase subunit E1-alpha (PDH E1α) as mitochondrial (M) markers, GAPDH as a cytosolic marker (C), and histone 3 (H3) as a nuclear marker (N). Blots originate from independent runs. (**I**) Representative immunoblots showing NTHL1 and OXPHOS protein levels in WT and *NTHL1^−/−^* cells complemented with NTHL1-RFP or the NTHL1(Δ21–60)-RFP deletion mutant. Tubulin serves as the loading control. (**J,K**) Analysis of mitochondrial mtDNA-CN (*n* = 3) (J) and relative OXPHOS protein expression (*n* = 4) (K) in WT and *NTHL1^−/−^* cells following reintroduction of NTHL1 variants. Data are normalized to WT controls. Each dot represents an independent biological replicate. Error bars represent mean ± SD (*n* ≥ 2). *P ≤ .05; **P ≤ .01, one-way ANOVA.

To investigate the oxidative stress response associated with NTHL1 deficiency, cells were treated with the ROS scavenger NAC. NAC treatment effectively reversed mtDNA-CN (Fig. 6A) and OXPHOS protein levels to those observed in WT cells, indicating that reduction of ROS is sufficient to reverse the phenotype of *NTHL1^−/−^* cells (Fig. 6B and C). Parallel experiments using the mitochondrial-targeted ROS scavenger XJB-5-131 similarly rescued mtDNA content and OXPHOS levels (Fig. 6D–F), underscoring a central role for mitochondrial ROS in driving these adaptive responses.

We next reasoned that if NTHL1 function within the mitochondria is critical for the observed phenotype, selective reintroduction of NTHL1 to this compartment should be sufficient to rescue it. To test this hypothesis, we engineered a truncated NTHL1 variant lacking nuclear localization. The NTHL1 N-terminus harbors nuclear localization signals (NLS), including two basic amino acid clusters previously described as NLS-1 (residues 1–41) and NLS-2 (residues 45–52) [58]. Earlier studies demonstrated that deletion mutants Δ21–41 and Δ42–60 spanning these regions exhibit reduced, but not abolished, nuclear localization [13]. Using the NLStradamus prediction [59], we identified a unique sequence encompassing residues 28–55 that integrates both NLS-1 and NLS-2 and is predicted to mediate nuclear targeting (Fig. 6G). We therefore hypothesized that deletion of residues 21–60 would fully abrogate nuclear localization. *In silico* analysis predicted that the Δ21–41 and Δ42–60 mutants would retain ~13% and 26% nuclear localization, respectively, whereas the Δ21–60 mutant would result in complete nuclear exclusion (Supplementary Fig. S7A). Subcellular fractionation confirmed that the NTHL1 Δ21–60 deletion mutant localizes to mitochondria and was not detectable in the nucleus compared to the NTHL1 full-length isoform 1 (FL) (Fig. 6H). Importantly, reintroduction of either FL NTHL1 or the Δ21–60 mutant restored DNA repair activity in *NTHL1^−/−^* cells, confirming the enzymatic proficiency of both variants (Supplementary Fig. S7B–D). To directly assess the relative functional contributions of mitochondrial and nuclear NTHL1, we performed rescue experiments using FL or the mitochondrial-restricted NTHL1 variant. Complementation of either variant restored mtDNA-CN and OXPHOS protein levels in NTHL1-deficient cells (Fig. 6I–K), demonstrating that loss of mitochondrial NTHL1 is the primary driver of the observed phenotype.

Taken together, these findings support a model in which NTHL1 deficiency elicits ROS-dependent signaling that promotes mitochondrial adaptation. Both mitochondrial ROS modulation and restoration of mitochondrial NTHL1 converge to normalize mtDNA content and respiratory function, revealing a coordinated adaptive stress response that originates from mitochondria.

### Loss of NTHL1 activates a non-canonical stress response

**Figure 7.**
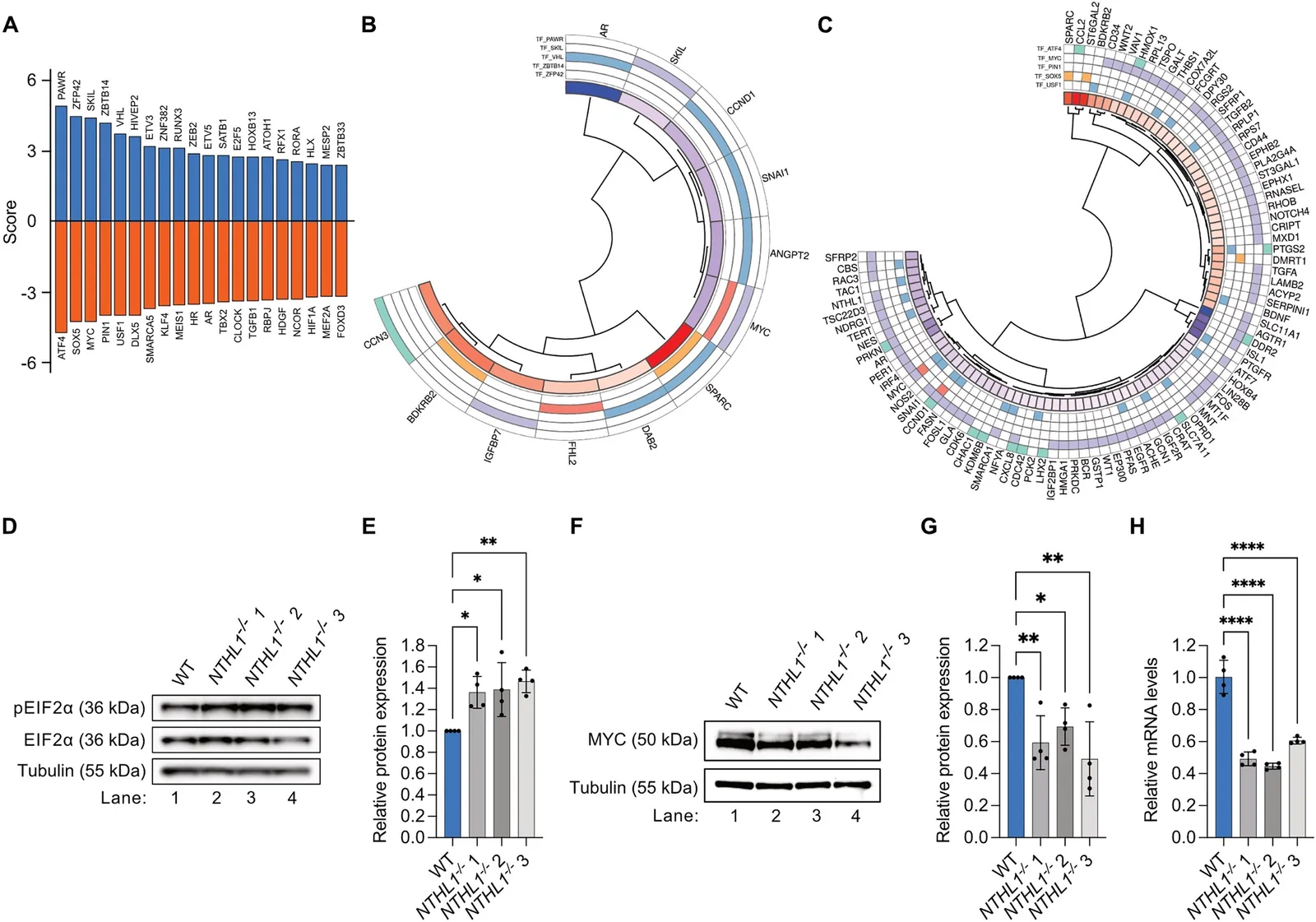
Loss of NTHL1 activates non-canonical stress response: (**A**) TF activity inference in *NTHL1*^−/−^ cells using decoupleR with the OmniPath regulatory network. Shown are the top 20 TFs with positive and negative activity scores. DEGs associated with the five highest-scoring positive TFs (**B**) and the five highest-scoring negative TFs (**C**). Immunoblot analysis (**D**) and quantification (**E**) of phosphorylated eIF2α (p-eIF2α) and total eIF2α in WT and *NTHL1^−/−^* cells (*n* = 4). Immunoblot analysis (**F**) and quantification of MYC protein (**G**) and *MYC* mRNA levels (**H**) in WT and *NTHL1^−/−^* cells (*n* = 4). Each dot represents an independent biological replicate. Error bars represent mean ± SD (*n* ≥ 3). *P ≤ .05; **P ≤ .01; ^****^P ≤ .0001, one-way ANOVA.

To identify transcriptional regulators underlying the phenotype observed in *NTHL1^−/−^* cells, we performed TF activity inference, ranking the top 20 TFs with the highest positive activity scores and negative activity scores (Fig. 7A). We then examined significant DEGs associated with the five highest-scoring positive TFs (Fig. 7B) and the five highest-scoring negative TFs (Fig. 7C). Positive-scoring TFs were associated with relatively few target genes, suggesting limited transcriptional impact. In contrast, negative-scoring TFs were linked to substantially larger gene sets, indicating broader regulatory influence. Among these, ATF4 and MYC ranked as the top negative-scoring TFs (first and third, respectively) and were associated with the highest number of regulated genes.

ATF4 is a central effector of the ISR, acting downstream of phosphorylated eIF2α to coordinate adaptive transcriptional programs that maintain redox balance, metabolism, and mitochondrial function [60]. In *NTHL1^−/−^* cells, several genes whose transcriptional activation is dependent on ATF4 were upregulated, including CCL2, HMOX1, and PTGS2, which are associated with metabolic stress, oxidative stress, and inflammatory signaling [61]. Conversely, some canonical ATF4 targets, such as DDR2 and SLC7A11, were downregulated, indicating a transcriptional stress response that diverges from the classical ISR. The upregulation of HMOX1, CCL2, and PTGS2 supports ISR activation, as HMOX1 is an ATF4-responsive oxidative stress gene [62], and CCL2 and PTGS2 act downstream of ATF4 signaling in ER stress–associated inflammation [63, 64]. To assess whether ISR was changed by NTHL1 loss, we evaluated eIF2α phosphorylation. Immunoblot analysis revealed significantly increased phosphorylated eIF2α levels in *NTHL1^−/−^* cells (Fig. 7D and E), confirming ISR activation despite the negative ATF4 activity score, suggesting a complex transcriptional regulation likely reflecting the convergenceof different transcriptional responses.

MYC, a key regulator of cell growth, metabolism, and mitochondrial biology [65], also scored as a negative TF and controlled the majority of the deregulated genes. *MYC* expression and protein levels were downregulated in *NTHL1^−/−^* cells (Fig. 7F–H). The combined alterations in ATF4- and MYC-regulated genes may indicate coordinated transcriptional reprogramming that balances stress adaptation with metabolic remodeling.

Collectively, these findings demonstrate that NTHL1 loss triggers a non-canonical stress response that can be explained by partial ATF4-dependent ISR activation and transcriptional reprogramming, alongside broader MYC pathway alterations. This complex transcriptional phenotype likely contributes to mitochondrial adaptation and altered OXPHOS capacity, linking NTHL1 deficiency to both stress response modulation and metabolic remodeling.

## Discussion

Here, we provide new insight into the interplay between DNA repair and mitochondrial regulation, a relationship that remains poorly understood. While NTHL1 has been reported to localize to mitochondria [66], its functional role in this organelle has remained elusive. Our study demonstrates that NTHL1 has a critical role in mitochondria, where its loss alters the transcript levels of mitochondrial genes. This extends previous reports that DNA glycosylases can influence transcription in the nucleus [41, 67, 68].

Unexpectedly, despite the accumulation of mtDNA lesions in NTHL1-deficient cells (Fig. 3A–D), we observed an increase in mtDNA-CN. This dissociation between lesion burden and copy number challenges the view that mtDNA instability inevitably leads to genome depletion [6, 69–71], and points instead to a compensatory response. One possible explanation involves changes in the TFAM/mtDNA ratio, which regulates the compaction and accessibility of mtDNA both *in vitro* [72] and in cells [73]. The observed mtDNA damage, coupled with reduced TFAM binding, suggests that certain DNA lesions may hinder TFAM–DNA interactions, potentially relaxing nucleoid compaction and allowing enhanced replication.

At this stage, we cannot determine whether the observed reduction in TFAM binding is a downstream consequence of the increased mtDNA content—potentially reflecting a dilution of TFAM relative to a more abundant genome—or whether NTHL1-specific DNA lesions actively facilitate mtDNA replication by promoting nucleoid decompaction associated with reduced TFAM binding. Distinguishing between these possibilities is critical for understanding the directionality of the relationship between TFAM dynamics and mitochondrial genome expansion. Future studies should therefore address causality, particularly in light of the uneven distribution of TFAM along mtDNA [46], which depends on sequence-specific TFAM–DNA binding interactions [74], cooperative TFAM–TFAM binding [75], and mtDNA compaction mechanisms [72]. Moreover, TFAM’s emerging role in DNA repair warrants consideration. TFAM has been shown to process AP sites [76] and to modulate the activity of monofunctional glycosylases such as UNG and OGG1 [77]. Its potential interaction with bifunctional glycosylases like NTHL1 remains unclear. Since bifunctional glycosylases can independently process AP sites, TFAM and NTHL1 may play opposing roles in mtDNA maintenance.

Further, we showed that *NTHL1^−/−^* cells exhibited increased mitochondrial mass, elevated levels of the mitochondrial fusion protein OPA1, and enhanced respiratory efficiency (Figs 3 and 4), suggesting mitochondrial network remodeling deriving from increased mtDNA-CN. These findings align with previous studies using cell models with stably reduced mtDNA-CN, which correlated with decreased mitochondrial protein expression and a decline in respiratory enzyme activity [78]. Furthermore, *NTHL1*^−/−^ cells exhibited increased tolerance to mitochondrial stress, particularly in response to complex I NADH oxidoreductase inhibition by MPP^+^ [52, 55–57] (Fig. 5), revealing a beneficial functional adaptation. This effect is most likely driven by the increased mitochondria content and increased OXPHOS levels. Although DNA glycosylase deficiency has not been directly linked to MPP^+^ resistance in humans, Sanders *et al*. demonstrated that functional variants of the BER genes modify PD risk in the context of oxidative-stress-related pesticide exposure, including paraquat, which is associated with mitochondrial function [79]. This role of NTHL1 is conserved, as our results align with observations in a *C. elegans* PD model in which *nth-1* mutants exhibit similar resistance to mitochondrial stressors [10].

Our findings suggest that loss of NTHL1 triggers compensatory mechanisms aimed at preserving mitochondrial function under elevated oxidative stress. The observed increase in proton leak and non-mitochondrial oxygen consumption reflects persistent oxidative stress and implicates activation of ROS-generating pathways. The ROS dependence of this phenotype is supported by its reversal following treatment with either general or mitochondrial ROS scavengers (Fig. 6). These observations are consistent with previous reports demonstrating increased mitochondrial content and mtDNA as adaptive responses to oxidative stress in human cells [80]. The rapid normalization of mtDNA-CN following short-term ROS scavenger treatment suggests a dynamic adjustment to restored redox homeostasis, most likely driven by changes in mitochondrial biogenesis or turnover, rather than acute mtDNA degradation via an unidentified pathway.

Given the mitochondrial ROS dependence of the phenotype, we investigated the role of mitochondrially localized NTHL1. Although mitochondrial localization of NTHL1 has not been extensively characterized in humans and may differ from mouse NTHL1 due to a stronger mitochondrial targeting sequence in mice [58], NTHL1 is known to be a dually targeted protein. Accordingly, we removed the N-terminal NLS, thereby directing NTHL1 to the mitochondria, which was sufficient to restore mtDNA-CN and OXPHOS levels to those of WT cells (Fig. 6 and Supplementary Fig. S7). Together, these results confirm that the observed phenotype originates from mitochondria.

Ultimately, the interaction of NTHL1 with the ISR and MYC-dependent transcriptional pathways introducesa new perspective on how mitochondrial stress is managed at the cellular level. Extensive studies using MYC-overexpressing or MYC-deficient cellular models have demonstrated that MYC protein directly regulates the transcription of nuclear genes encoding mitochondrial proteins, thereby acting as a central driver of mitochondrial biogenesis and function [81–83] (reviewed in [65]). Li *et al*. showed using a human lymphoblastoid cell line that MYC overexpression leads to increased mitochondrial mass, whereas *MYC* depletion produces the opposite effect [81]. In our system, MYC expression is reduced, possibly reflecting a feedback response to the already elevated mitochondrial mass observed following NTHL1 loss. Such downregulation may represent a homeostatic mechanism to restrain further mitochondrial biogenesis under conditions of sustained mitochondrial stress, thereby linking NTHL1 deficiency to ISR changes and modulation of MYC-driven transcriptional control.

Collectively, our findings support the concept of mitohormesis, in which mild mitochondrial stress elicits adaptive responses that enhance cellular resilience. Mitochondrial stress also extends beyond the organelle itself, influencing cytosolic proteostasis by modulating protein synthesis, stability, and degradation to sustain metabolism and proliferation. In this context, NTHL1 deficiency appears to reprogram these interconnected networks, shifting the cellular response toward stress adaptation rather than overt dysfunction. This highlights an intricate crosstalk between DNA repair, mitochondrial homeostasis, and cellular adaptation, with potential relevance to neurodegeneration, aging, and cancer. Consistent with previous studies, we observe upregulation of mitochondrial biogenesis–associated genes and activation of stress response pathways, reflecting a common compensatory response to mitochondrial impairment.

An unresolved question concerns the long-term consequences of NTHL1 deficiency. While compensatory mechanisms may initially preserve mitochondrial function, sustained activation of these pathways could ultimately promote metabolic imbalance or oxidative stress, contributing to cellular aging or pathology. Although our findings are consistent with a neuroprotective mitohormesis phenotype described in *C. elegans*, a mammalian *in vivo* model would extend the conclusions regarding organismal aging, tissue-specific effects, and long-term consequences of NTHL1 loss on mitochondrial health and disease susceptibility.

Overall, our work reveals previously unappreciated connections between mtDNA quality control and cellular stress responses, and identifies NTHL1 as a potential regulator of mitochondrial homeostasis. Further investigation will be necessary to define its broader impact on cellular physiology and disease.

## Supplementary Material

gkag876_Supplemental_File

## Data Availability

Raw files (FASTQ) and processed counts associated with RNA sequencing data presented in Fig. 2 are available in GEO under accession number GSE291371.
