# Supplementary material for: Functional impact of Nth like DNA glycosylase 1 on mitochondrial dynamics
Source: Nucleic Acids Res. 2026 Sep 15;54(17):gkag876. doi: 10.1093/nar/gkag876 (PMC13575422; doi:10.1093/nar/gkag876)
Supplement: Supplementary file 1 [file gkag876_supplemental_file.pdf]

SUPPLEMENTARY DATA

Figure S1

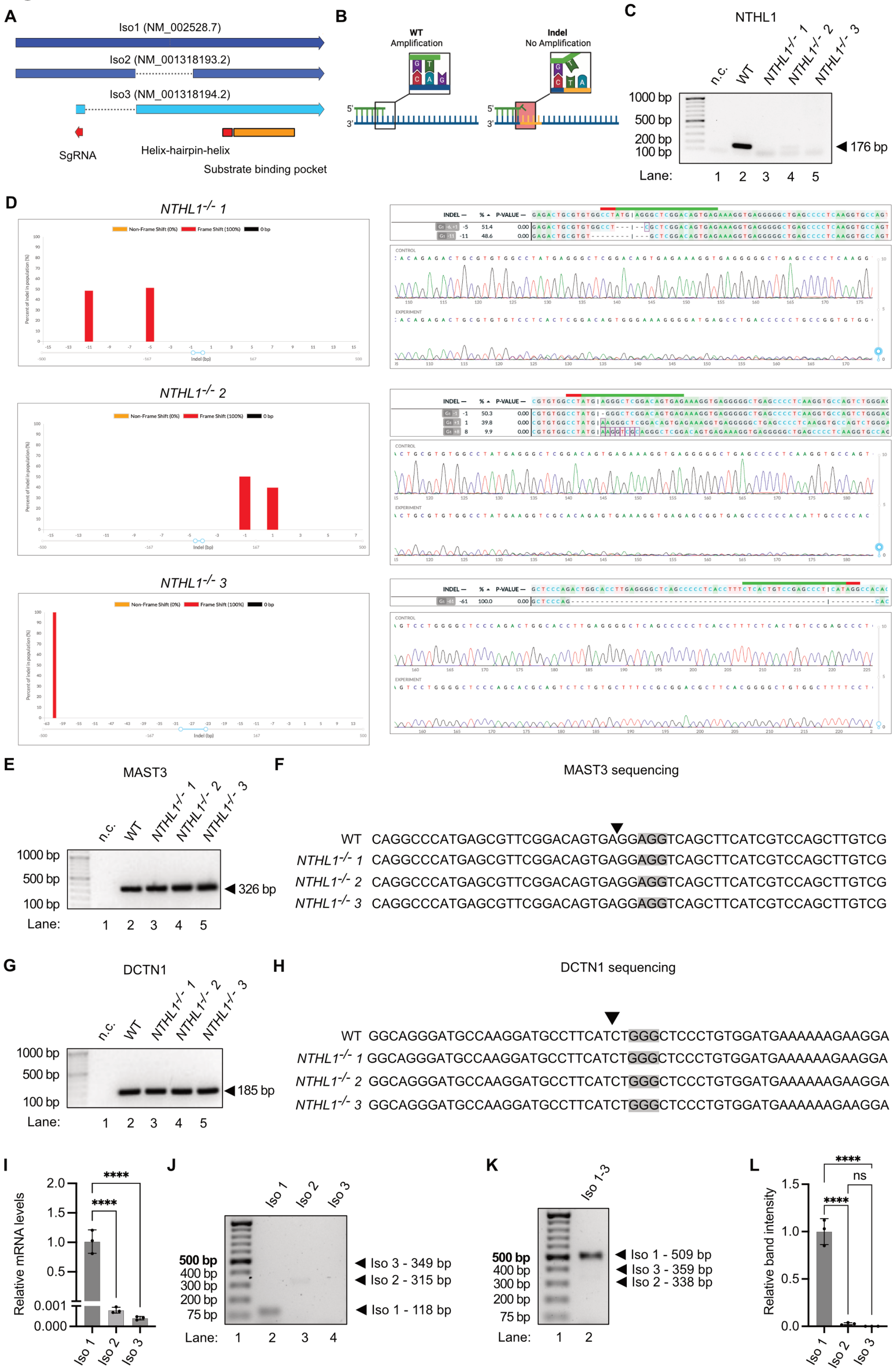

Figure S1: Screening and quality control of *NTHL1*$^{-/-}$ clones. (A) Schematic representation of the CRISPR-Cas9 design. The cut site (red) is shown in relation to the exons (blue) on all three isoforms of NTHL1. Since the cut is upstream of the substrate-binding pocket, the resulting protein is non-functional. **(B)** Indel-specific PCR: An indel-specific PCR was performed to screen for mutations in NTHL1 knockout clonal cell lines. The PCR does not yield a product in the presence of indel mutations, confirming successful targeting. **(C)** Clonal screening: Individual clonal cell lines were isolated and screened for indel mutations in the sgRNA-targeted region of *NTHL1* using indel-specific PCR, confirming gene disruption at the DNA level. **(D)** Sanger sequencing validation of biallelic indel mutations: Indel mutations in selected knockout clones were validated by Sanger sequencing. Chromatograms of PCR-amplified CRISPR target regions were analyzed to characterize biallelic indel mutations, confirming precise genome editing by CRISPR/Cas9. **(E-H)** Off-target analysis: To ensure the specificity of the CRISPR/Cas9 system, potential off-target sites identified by CRISPOR were examined. Exonic off-target regions in MAST3 **(E)** and DCTN1 **(G)** were screened using indel-specific PCR, and the amplified regions were further confirmed to be free of mutations by Sanger sequencing **(F, H)**. **(I)** RTqPCR analysis of NTHL1 isoforms. Isoform 1 exhibited the highest expression levels, while isoforms 2 and 3 were detected at lower levels. **(J)** gel electrophoresis of PCR using specific primers targeting each individual isoform. **(K)** gel electrophoresis PCR using primers able of amplifying all three isoforms. Isoform 1 exhibited the highest expression levels, while isoforms 2 and 3 were detected at lower levels. **(L)** Quantification of experiment depicted in K.

Figure S2

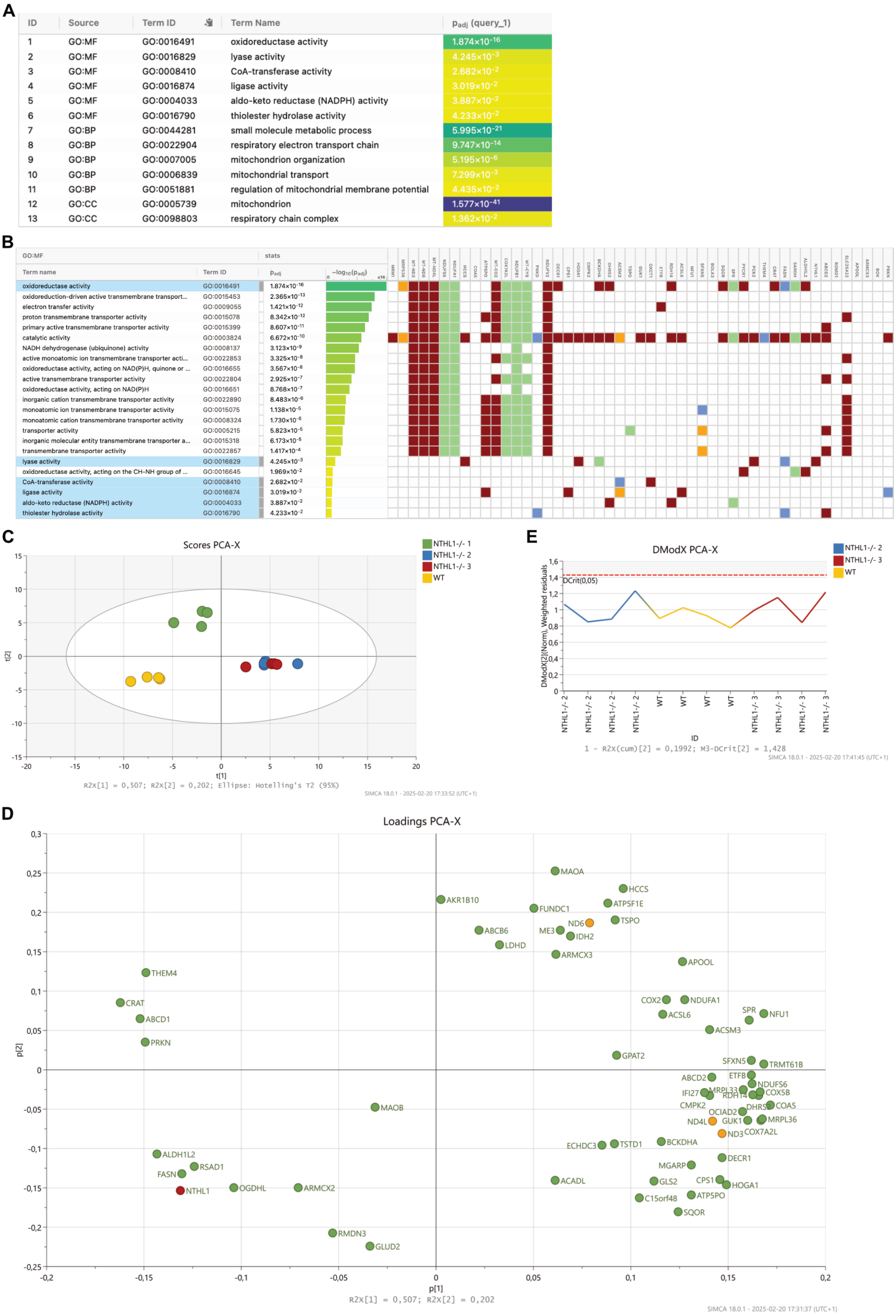

A

ID	Source	Term ID	Term Name	p_{adj} (query_1)
1	GO:MF	GO:0016491	oxidoreductase activity	1.874×10^{-16}
2	GO:MF	GO:0016829	lyase activity	4.245×10^{-3}
3	GO:MF	GO:0008410	CoA-transferase activity	2.682×10^{-2}
4	GO:MF	GO:0016874	ligase activity	3.019×10^{-2}
5	GO:MF	GO:0004033	aldo-keto reductase (NADPH) activity	3.887×10^{-2}
6	GO:MF	GO:0016790	thiolester hydrolase activity	4.233×10^{-2}
7	GO:BP	GO:0044281	small molecule metabolic process	5.995×10^{-21}
8	GO:BP	GO:0022904	respiratory electron transport chain	9.747×10^{-14}
9	GO:BP	GO:0007005	mitochondrion organization	5.195×10^{-6}
10	GO:BP	GO:0006839	mitochondrial transport	7.299×10^{-3}
11	GO:BP	GO:0051881	regulation of mitochondrial membrane potential	4.435×10^{-2}
12	GO:CC	GO:0005739	mitochondrion	1.577×10^{-41}
13	GO:CC	GO:0098803	respiratory chain complex	1.362×10^{-2}

Figure S2: GO Enrichment and PCA analysis of differentially expressed mitochondrial genes in *NTHL1$^{-/-}$* and WT samples. **(A)** GO Enrichment Analysis: top significantly enriched terms for molecular function (MF), biological process (BP), cellular component (CC) and their adjusted p-value ($-\log_{10}$ Padj). **(B)** GO Enrichment analysis of the top significantly enriched MF terms. The bar chart illustrates the gene set's primary contributions to key MF, with each bar representing a distinct GO category. Color intensity corresponds to statistical significance (adjusted p-value). **(C)** Score plot showing clear separation between *NTHL1$^{-/-}$* 1 (green), 2 (blue), 3 (red) and WT (yellow) samples along the first predictive component (t[1]), indicating distinct expression profiles of mitochondrial genes between the two groups. **(D)** Loadings plot identifying the contributions of individual genes to the separation between KO and WT samples. **(E)** DModX line plot indicating no significant outliers in the dataset, with all samples falling below the critical threshold (DCrit = 0.05), confirming the robustness of the analysis.

Figure S3

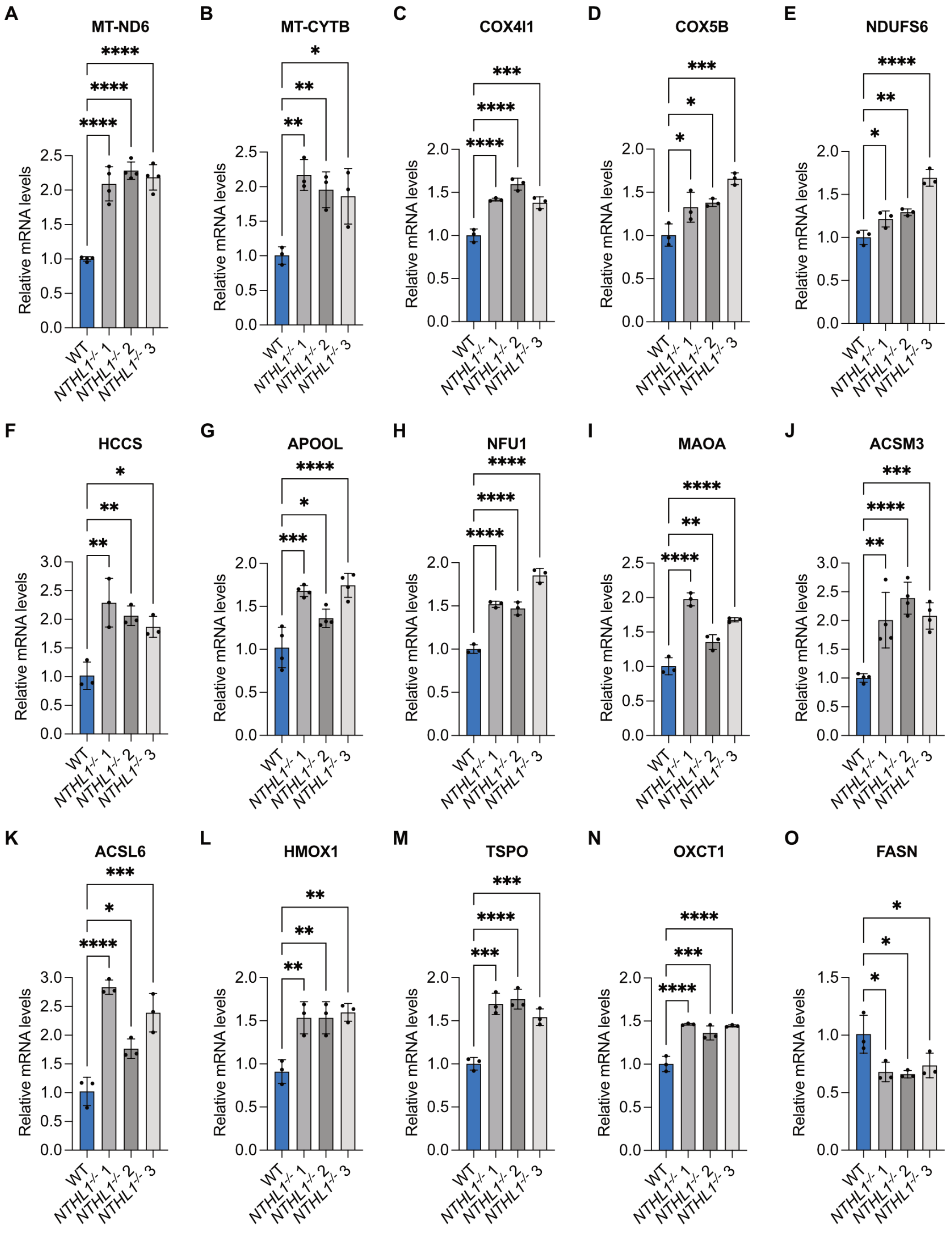

Figure S3: RT-qPCR validation of RNA-Seq data. RT-qPCR analysis confirms the differential expression of selected genes *MT-ND6* **(A)**, *MT-CYTB* **(B)**, *COX4I1* **(C)**, *COX5B* **(D)**, *NDUFS6* **(E)**, *HCCS* **(F)**, *APOOL* **(G)**, *NFU1* **(H)**, *MAOA* **(I)**, *ACSM3* **(J)**, *ACSL6* **(K)**, *HMOX1* **(L)**, *TSPO* **(M)**, *OXCT1* **(N)**, *FASN* **(O)**. Data are represented as fold change in *NTHL1*$^{-/-}$ cells relative to WT cells. Error bars represent mean ± SD (n ≥ 3). *p ≤ 0.05; **p ≤ 0.01; ***p ≤ 0.001; ****p ≤ 0.0001, one-way ANOVA.

Figure S4

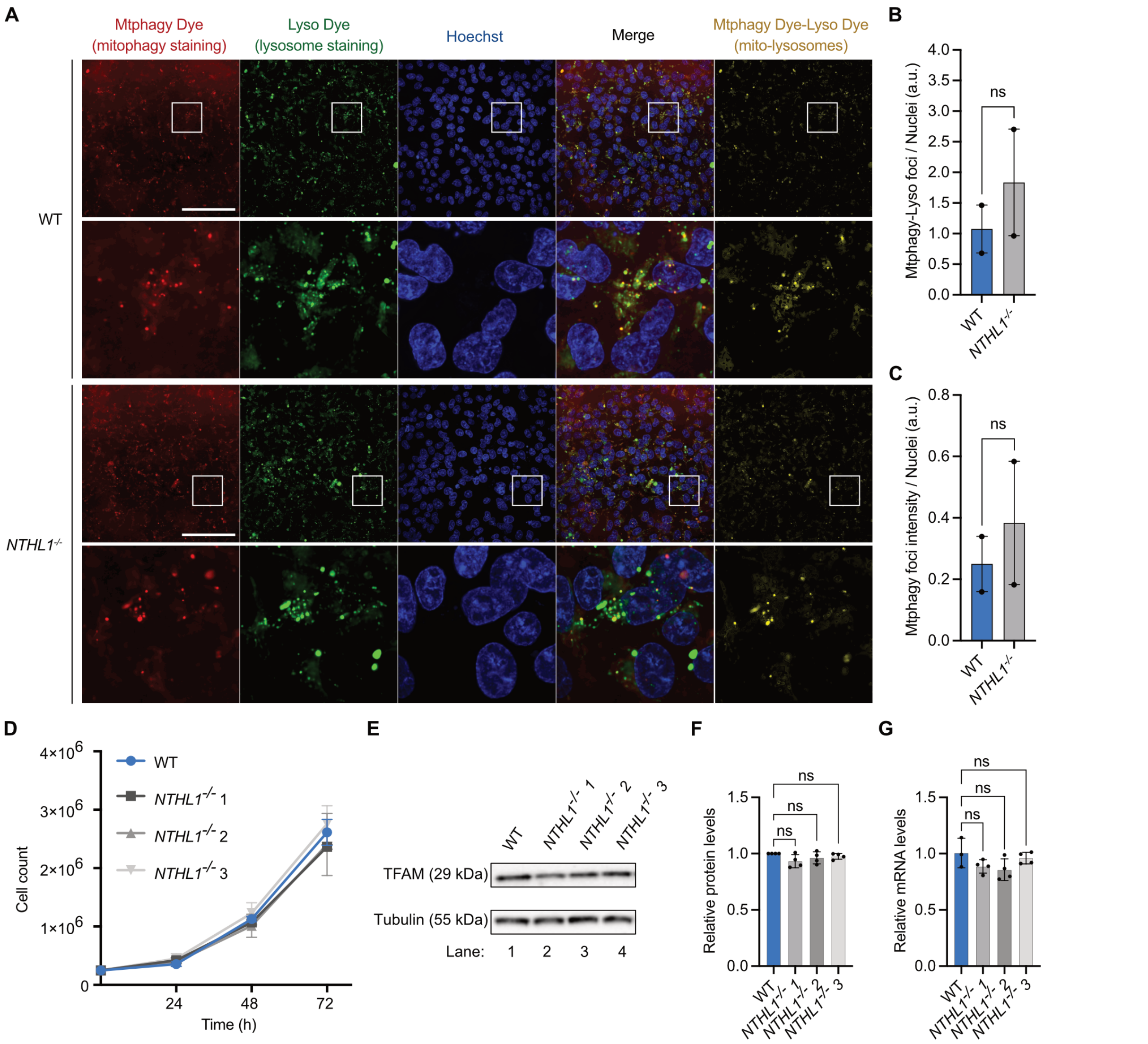

Figure S4: Cell proliferation, mitophagy and TFAM levels in *NTHL1$^{-/-}$* cell Lines. (A-C) Assessment of basal mitophagy in WT and *NTHL1$^{-/-}$* cells. **(A)** Representative images showing fluorescence intensity of Mtphagy Dye and Lyso Dye and their colocalization, indicating mitophagy–lysosome association. Nuclei are stained with Hoechst (blue). Scale bar, 100 µm. **(B)** Quantification of number of co-localizing of mitophagy-lysosomes colocalization events (mito-lysosomes) per cell. **(C)** Quantification of the average fluorescence intensity of Mtphagy Dye foci per cell. Error bars represent mean ± SEM (n = 2). T-test. ns – not significant. **(D)** Cell proliferation analysis of WT and *NTHL1$^{-/-}$* cells seeded at equal densities and counted over a 72-h period. **(E)** Immunoblot analysis of TFAM in extracts from HEK293 WT (lane 1) and 3 independent HEK293 *NTHL1$^{-/-}$* clones (lanes 2-4). **(F)** Quantification of the data shown in panel E. **(G)** RT-qPCR Validation of RNA-seq Data: RT-qPCR analysis confirms no differential expression of selected TFAM gene. Data are represented as fold change in *NTHL1$^{-/-}$* cells relative to WT cells. Error bars represent mean ± SD (n ≥ 3). one-way ANOVA. ns – not significant.

Figure S5

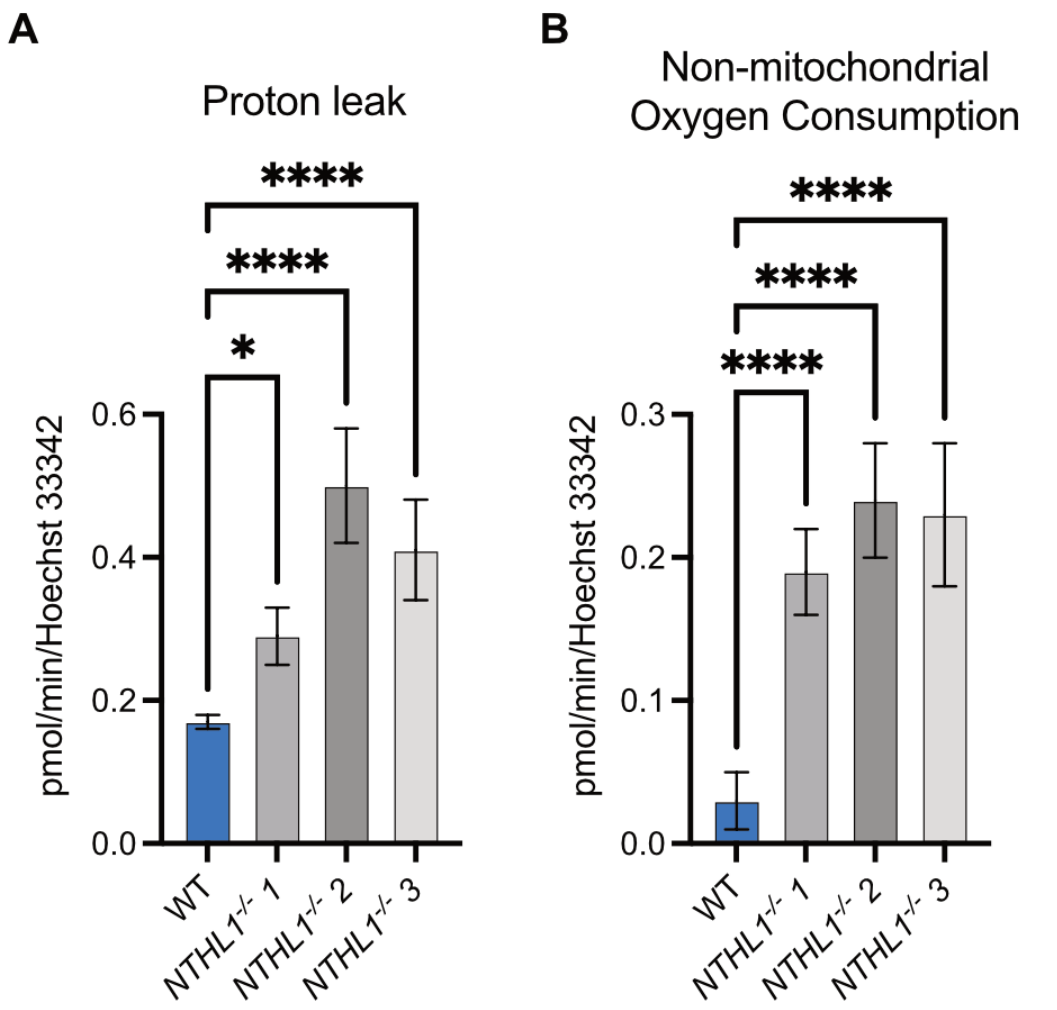

Figure S5: Increased proton leak and non-mitochondrial oxygen consumption in NTHL1-deficient cell lines. (A) Proton Leak: NTHL1-deficient cells show increased proton leak, indicating energy dissipation as heat instead of ATP production, suggesting altered mitochondrial efficiency. **(B)** Non-Mitochondrial Oxygen Consumption: These cells also exhibit increased non-mitochondrial oxygen consumption, reflecting heightened oxidative activity and potential ROS generation outside the mitochondria.

Figure S6

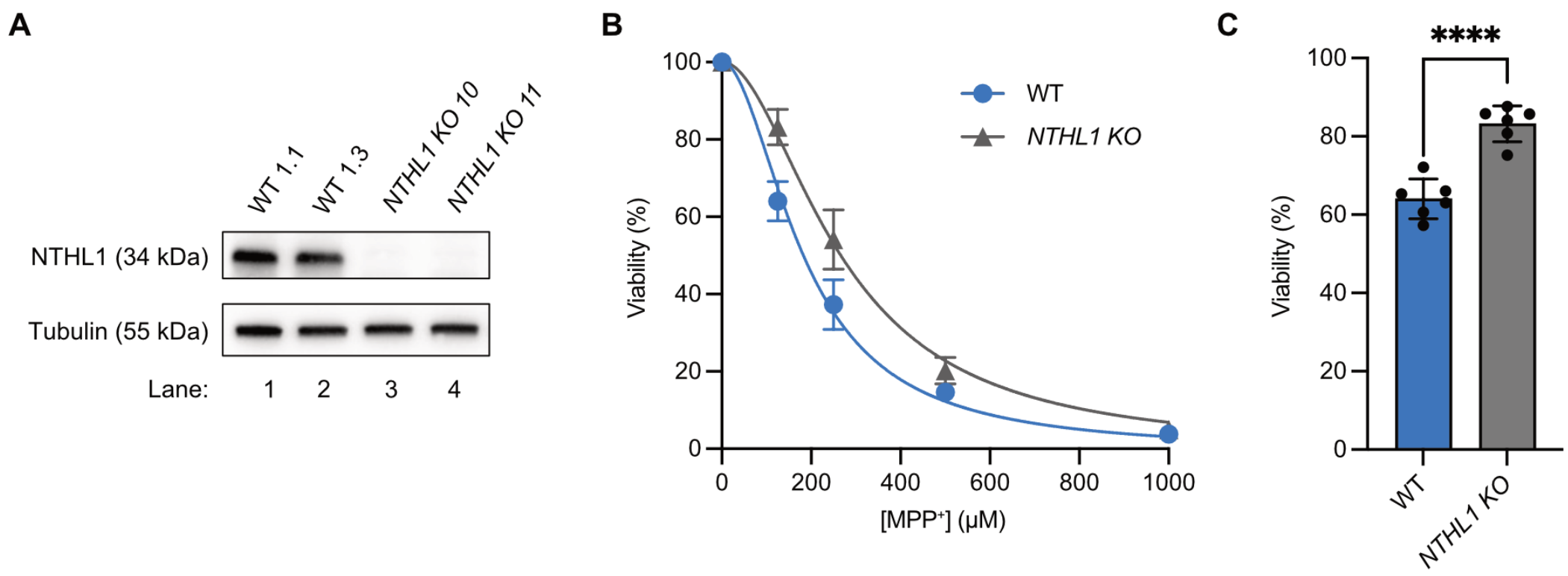

Figure S6: NTHL1-deficient HAP1 cells display enhanced tolerance to MPP$^+$. (A) Immunoblot analysis of NTHL1 in extracts from HAP1 WT (lane 1, 2) and 2 independent HAP1 NTHL1 KO clones (lanes 3, 4). **(B)** Viability of HAP1 WT and NTHL1 KO cell lines after 72h treatment with indicated amounts of MPP$^+$. **(C)** Viability of HAP1 WT and NTHL1 KO cell lines after 72h treatment with indicated 125 µM MPP$^+$. Data are represented as fold change in NTHL1 KO cells relative to WT cells. Error bars represent mean ± SD (n ≥ 3). ****p ≤ 0.0001, t-test.

Figure S7

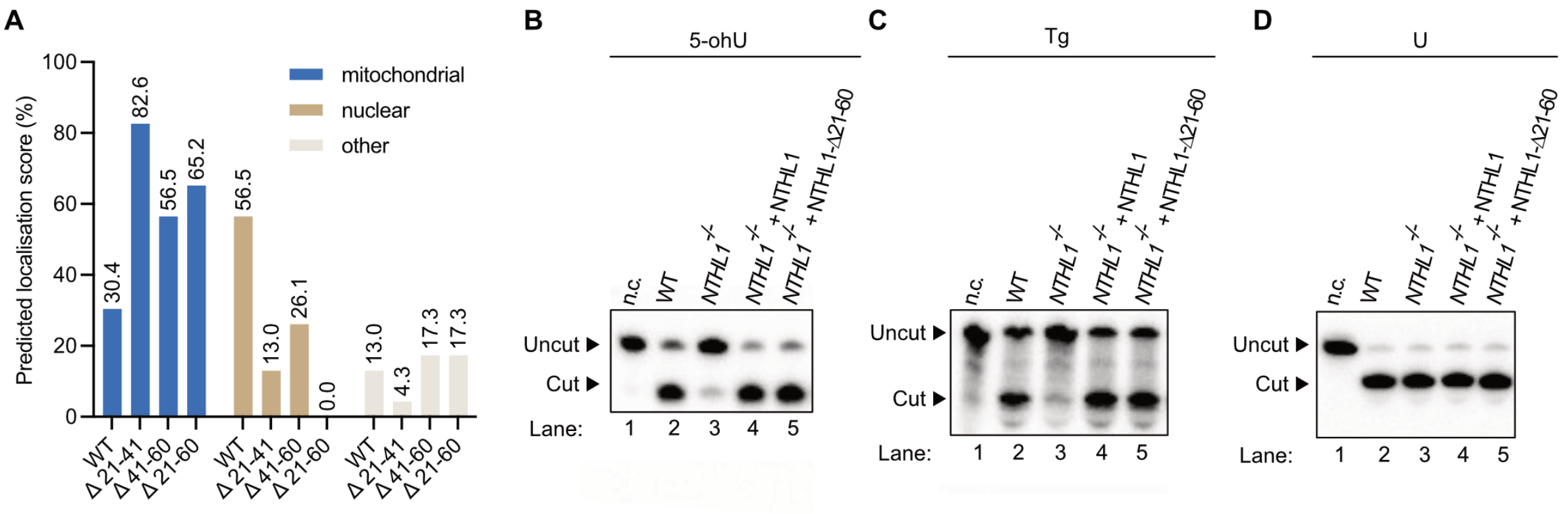

Figure S7: The NTHL1 Δ21–60 deletion mutant is predicted to be mitochondrial, excluded from the nucleus, and enzymatically active. (A) Predicted subcellular localization of NTHL1 truncation mutants based on PSORT II analysis. **(B-D)** DNA glycosylase activity assay: No-extract negative control (n.c.; lane 1) or 10 µg WCE from HEK293 WT cells (lane 2), *NTHL1*$^{-/-}$ cells (lane 3) transfected with control plasmids or expressing NTHL1 iso1-RFP (lane 4) or the NTHL1 iso1-RFP Δ21-60 deletion mutant (lane 5) on ^{32}P-end-labelled double-stranded oligonucleotides containing 5-hydroxyuracil (5-ohU) **(B)**, Thymine glycol (Tg) **(C)** and Uracil (U) **(D)**.

Supplementary Table 1. Gene names and gene identifiers of differentially expressed genes identified in *NTHL1*$^{-/-}$ cells used for GO molecular function analysis in Figure 2.

**Upregulated**	
**gene_name**	**gene_id**
CD99	ENSG00000002586
HECW1	ENSG00000002746
ARX	ENSG00000004848
HCCS	ENSG00000004961
WDR54	ENSG00000005448
TBXA2R	ENSG00000006638
TRAPPC6A	ENSG00000007255
IL32	ENSG00000008517
SEMA3G	ENSG00000010319
SNAI2	ENSG00000019549
CPS1	ENSG00000021826
TMSB10	ENSG00000034510
GPM6B	ENSG00000046653
XK	ENSG00000047597
ANO2	ENSG00000047617
CC2D2A	ENSG00000048342
CYBA	ENSG00000051523
ATP2B4	ENSG00000058668
MXD1	ENSG00000059728
ATP2B3	ENSG00000067842
REEP1	ENSG00000068615
KCNAB2	ENSG00000069424
RPL31	ENSG00000071082
PTGS2	ENSG00000073756
MAP2	ENSG00000078018
COL16A1	ENSG00000084636
MAPRE3	ENSG00000084764
ERO1B	ENSG00000086619
MMP2	ENSG00000087245
TMEM40	ENSG00000088726
SEC14L2	ENSG00000100003
DHRS2	ENSG00000100867
FERMT1	ENSG00000101311
SYNDIG1	ENSG00000101463
STS	ENSG00000101846
SYP	ENSG00000102003
PCSK1N	ENSG00000102109
TIMP1	ENSG00000102265
BEX4	ENSG00000102409
NALCN	ENSG00000102452
DECR1	ENSG00000104325
NIPAL2	ENSG00000104361
LIN7B	ENSG00000104863
PEX11G	ENSG00000104883
ETFB	ENSG00000105379
MEIS3	ENSG00000105419
CNFN	ENSG00000105427
CLEC11A	ENSG00000105472
TMEM59L	ENSG00000105696
HSPB1	ENSG00000106211

NPTX2	ENSG00000106236
SFRP4	ENSG00000106483
TMEM176B	ENSG00000106565
CCL2	ENSG00000108691
CPE	ENSG00000109472
B3GAT1	ENSG00000109956
FOLR1	ENSG00000110195
NEDD9	ENSG00000111859
SPARC	ENSG00000113140
UNC5A	ENSG00000113763
ACTR1B	ENSG00000115073
TP53I3	ENSG00000115129
SLC30A3	ENSG00000115194
FNDC4	ENSG00000115226
POLE4	ENSG00000115350
EVA1A	ENSG00000115363
EFEMP1	ENSG00000115380
UNC50	ENSG00000115446
FHL2	ENSG00000115641
QPCT	ENSG00000115828
LGALS8	ENSG00000116977
RGS4	ENSG00000117152
CDK18	ENSG00000117266
IRF6	ENSG00000117595
NENF	ENSG00000117691
YPEL5	ENSG00000119801
YIPF4	ENSG00000119820
SFRP5	ENSG00000120057
NPY	ENSG00000122585
NCKAP1L	ENSG00000123338
PAIP2B	ENSG00000124374
TMEM255A	ENSG00000125355
NDUFA1	ENSG00000125356
BFSP1	ENSG00000125864
MMP24	ENSG00000125966
IFI6	ENSG00000126709
PIN1	ENSG00000127445
GNG11	ENSG00000127920
GNGT1	ENSG00000127928
SPINK2	ENSG00000128040
APOC1	ENSG00000130208
PXDN	ENSG00000130508
CCT6B	ENSG00000132141
SLC6A11	ENSG00000132164
EFR3A	ENSG00000132294
CARD6	ENSG00000132357
GPR12	ENSG00000132975
TRPC4	ENSG00000133107
BEX2	ENSG00000133134
TCEAL4	ENSG00000133142
BEX1	ENSG00000133169
NTS	ENSG00000133636
NGF	ENSG00000134259
CMPK2	ENSG00000134326

ANXA1	ENSG00000135046
PRADC1	ENSG00000135617
EMX1	ENSG00000135638
SP110	ENSG00000135899
COX5B	ENSG00000135940
CCN3	ENSG00000136999
DMRT1	ENSG00000137090
SYTL2	ENSG00000137501
CGREF1	ENSG00000138028
SLC40A1	ENSG00000138449
C1RL	ENSG00000139178
ADAM21	ENSG00000139985
MCTP2	ENSG00000140563
CHST4	ENSG00000140835
NLRC5	ENSG00000140853
DEF8	ENSG00000140995
GALNS	ENSG00000141012
ABCA8	ENSG00000141338
VAV1	ENSG00000141968
SYTL1	ENSG00000142765
RGL1	ENSG00000143344
SYT14	ENSG00000143469
KCNH1	ENSG00000143473
DUSP10	ENSG00000143507
CNIH3	ENSG00000143786
OSR1	ENSG00000143867
CHAC2	ENSG00000143942
RPS27A	ENSG00000143947
SNRPG	ENSG00000143977
ABHD1	ENSG00000143994
ST6GAL2	ENSG00000144057
FAHD2B	ENSG00000144199
NXPH2	ENSG00000144227
STXBP5L	ENSG00000145087
OCIAD2	ENSG00000145247
NDUFS6	ENSG00000145494
RPL37	ENSG00000145592
PLK2	ENSG00000145632
SHISAL2B	ENSG00000145642
ANKRD31	ENSG00000145700
LANCL3	ENSG00000147036
SYTL5	ENSG00000147041
CHST7	ENSG00000147119
IDNK	ENSG00000148057
HNMT	ENSG00000150540
ASTN1	ENSG00000152092
HSPB8	ENSG00000152137
PLEKHH2	ENSG00000152527
C16orf89	ENSG00000153446
PGCKA1	ENSG00000154274
WNT3A	ENSG00000154342
KCNMA1	ENSG00000156113
GALNT14	ENSG00000158089
CSRP1	ENSG00000159176

IRX6	ENSG00000159387
ABCG1	ENSG00000160179
AZGP1	ENSG00000160862
HAAO	ENSG00000162882
SLC66A3	ENSG00000162976
PRORSD1P	ENSG00000162997
VSNL1	ENSG00000163032
TGFA	ENSG00000163235
INAVA	ENSG00000163362
LINC01116	ENSG00000163364
LMOD1	ENSG00000163431
IGFBP7	ENSG00000163453
SERPINI1	ENSG00000163536
SMIM43	ENSG00000164112
TLR3	ENSG00000164342
CYBB	ENSG00000165168
DYNLT3	ENSG00000165169
PCDH19	ENSG00000165194
PHYHIPL	ENSG00000165443
FAAH2	ENSG00000165591
OXGR1	ENSG00000165621
FBN1	ENSG00000166147
LARP6	ENSG00000166173
RIC3	ENSG00000166405
PRKCB	ENSG00000166501
BEAN1	ENSG00000166546
BEX3	ENSG00000166681
ZCCHC18	ENSG00000166707
BMERB1	ENSG00000166780
MESP1	ENSG00000166823
RBFOX3	ENSG00000167281
RPL13	ENSG00000167526
ZNF610	ENSG00000167554
SEMA6B	ENSG00000167680
TRANK1	ENSG00000168016
BDKRB2	ENSG00000168398
UGT3A2	ENSG00000168671
B3GALNT1	ENSG00000169255
NFU1	ENSG00000169599
TMEM266	ENSG00000169758
REPS2	ENSG00000169891
B3GNT2	ENSG00000170340
LRRN2	ENSG00000170382
ACYP2	ENSG00000170634
KCNS3	ENSG00000170745
NRTN	ENSG00000171119
MRPL36	ENSG00000171421
PTGER4	ENSG00000171522
NEUROD2	ENSG00000171532
MAP6	ENSG00000171533
VAT1L	ENSG00000171724
KRCC1	ENSG00000172086
SLFN12	ENSG00000172123
ARNT2	ENSG00000172379

ZNF738	ENSG00000172687
CYP7B1	ENSG00000172817
LCLAT1	ENSG00000172954
COMMD1	ENSG00000173163
ABCD2	ENSG00000173208
ADGRG2	ENSG00000173698
HAP1	ENSG00000173805
PHLDA3	ENSG00000174307
STARD6	ENSG00000174448
BTC	ENSG00000174808
LPL	ENSG00000175445
SFN	ENSG00000175793
CREG2	ENSG00000175874
EID2B	ENSG00000176401
SLCO3A1	ENSG00000176463
BDNF	ENSG00000176697
MAGEB6	ENSG00000176746
SCN4B	ENSG00000177098
NINJ2-AS1	ENSG00000177406
EFCAB13	ENSG00000178852
C14orf39	ENSG00000179008
CCDC89	ENSG00000179071
ATOH7	ENSG00000179774
NLRP11	ENSG00000179873
TCEAL8	ENSG00000180964
FAM181B	ENSG00000182103
XKRX	ENSG00000182489
RGS7	ENSG00000182901
ZNF320	ENSG00000182986
IGSF5	ENSG00000183067
COA5	ENSG00000183513
SLC35F3	ENSG00000183780
TTC32	ENSG00000183891
KCNK12	ENSG00000184261
SLIT3	ENSG00000184347
FAM110C	ENSG00000184731
PTRHD1	ENSG00000184924
SLC24A3	ENSG00000185052
TCEAL9	ENSG00000185222
NTF3	ENSG00000185652
LYG2	ENSG00000185674
IFITM1	ENSG00000185885
CYP4X1	ENSG00000186377
TRABD2A	ENSG00000186854
TMEM17	ENSG00000186889
GABRD	ENSG00000187730
RINL	ENSG00000187994
COL4A5	ENSG00000188153
SAXO2	ENSG00000188659
ZNF676	ENSG00000196109
TCEAL3	ENSG00000196507
SLC30A10	ENSG00000196660
ZNF418	ENSG00000196724
S100A2	ENSG00000196754

LAMB3	ENSG00000196878
H2BC26	ENSG00000196890
SCOC-AS1	ENSG00000196951
SMIM10L2B	ENSG00000196972
SLC6A17	ENSG00000197106
ZNF257	ENSG00000197134
NUP62CL	ENSG00000198088
QRICH1	ENSG00000198218
UBL5	ENSG00000198258
HYLS1	ENSG00000198331
FLVCR1-DT	ENSG00000198468
RYR2	ENSG00000198626
MT-ND6	ENSG00000198695
BRINP2	ENSG00000198797
MT-ND3	ENSG00000198840
CCDC152	ENSG00000198865
SHISA4	ENSG00000198892
VTRNA1-3	ENSG00000202515
ZNF525	ENSG00000203326
PDCD4-AS1	ENSG00000203497
C1orf53	ENSG00000203724
PTPN20	ENSG00000204179
SYCE1L	ENSG00000205078
TMSB4X	ENSG00000205542
CYS1	ENSG00000205795
HCP5	ENSG00000206337
MT-RNR2	ENSG00000210082
MT-TT	ENSG00000210195
EWSAT1	ENSG00000212766
MT-ND4L	ENSG00000212907
LBH	ENSG00000213626
LIPE-AS1	ENSG00000213904
ZNF90	ENSG00000213988
C19orf38	ENSG00000214212
EML6	ENSG00000214595
RPL13P12	ENSG00000215030
UBE2QL1	ENSG00000215218
HMSD	ENSG00000221887
C19orf73	ENSG00000221916
ZNF880	ENSG00000221923
RFPL4A	ENSG00000223638
C1GALT1C1L	ENSG00000223658
LGALS8-AS1	ENSG00000223776
ENSG00000224269	ENSG00000224269
SNRPGP15	ENSG00000224543
LINC01117	ENSG00000224577
RPS27AP16	ENSG00000224631
TAF1A-AS1	ENSG00000225265
ENSG00000226465	ENSG00000226465
LINC01918	ENSG00000226508
LINC00355	ENSG00000227674
OST4	ENSG00000228474
C2orf92	ENSG00000228486
PROX1-AS1	ENSG00000230461

ENSG00000230747	ENSG00000230747
STMND1	ENSG00000230873
LINC01508	ENSG00000231107
MAP4K3-DT	ENSG00000231312
IPO9-AS1	ENSG00000231871
DUX4L50	ENSG00000232815
CNIH3-AS2	ENSG00000233384
ZNF892	ENSG00000233757
RPS28	ENSG00000233927
EIF2AK3-DT	ENSG00000234028
ENSG00000234286	ENSG00000234286
LINC01426	ENSG00000234380
LNCTAM34A	ENSG00000234546
TGFBRAP1-AS1	ENSG00000235319
SNRPGP10	ENSG00000235363
PSMD14-DT	ENSG00000235724
COP1-DT	ENSG00000236021
TLX1NB	ENSG00000236311
BCYRN1	ENSG00000236824
ZNF295-AS1	ENSG00000237232
FAM88E	ENSG00000237357
ZNF737	ENSG00000237440
TIAM1-AS1	ENSG00000237594
SLC20A1-DT	ENSG00000237753
LINC01410	ENSG00000238113
LINC02944	ENSG00000238276
KMT2E-AS1	ENSG00000239569
PSMB9	ENSG00000240065
HOGA1	ENSG00000241935
LINC00960	ENSG00000242516
CCDC169	ENSG00000242715
MRPL33	ENSG00000243147
PCDHAC2	ENSG00000243232
LIFR-AS1	ENSG00000244968
IGFBP7-AS1	ENSG00000245067
LINC02226	ENSG00000245729
ZBED9-AS1	ENSG00000246350
H2AJ	ENSG00000246705
BAALC-AS1	ENSG00000247081
PCDHAC1	ENSG00000248383
ENSG00000248837	ENSG00000248837
LINC01234	ENSG00000249550
FER1L5	ENSG00000249715
PCDHA10	ENSG00000250120
FAM218A	ENSG00000250486
SELENOP	ENSG00000250722
ENSG00000251131	ENSG00000251131
MRPS30-DT	ENSG00000251141
SCARNA6	ENSG00000251791
SCARNA5	ENSG00000252010
SCARNA21	ENSG00000252835
VPS13B-DT	ENSG00000253948
TDH-AS1	ENSG00000255020
ADCY6-DT	ENSG00000257660

H2AZ2P1	ENSG00000258741
TMC3-AS1	ENSG00000259343
ARNT2-DT	ENSG00000259495
ST20-AS1	ENSG00000259642
FBN1-DT	ENSG00000259705
HEATR3-AS1	ENSG00000259843
ENSG00000259977	ENSG00000259977
MBTPS1-DT	ENSG00000260018
CRIM1-DT	ENSG00000260025
MRFAP1P1	ENSG00000260081
CARMAL	ENSG00000260123
SNHG19	ENSG00000260260
C16orf95	ENSG00000260456
ENSG00000260593	ENSG00000260593
PAPOLA-DT	ENSG00000260806
WFDC21P	ENSG00000261040
VPS9D1-AS1	ENSG00000261373
MAFTRR	ENSG00000261390
ENSG00000261519	ENSG00000261519
ENSG00000261526	ENSG00000261526
GREP1	ENSG00000262152
LINC01977	ENSG00000262772
LINC00667	ENSG00000263753
SNRPGP2	ENSG00000264350
LINC00526	ENSG00000264575
MIR3181	ENSG00000264947
RN7SL5P	ENSG00000265735
PCAT19	ENSG00000267107
ENSG00000267248	ENSG00000267248
SETBP1-DT	ENSG00000267414
ENSG00000267458	ENSG00000267458
RUNDC3A-AS1	ENSG00000267750
ENSG00000269388	ENSG00000269388
LINC02893	ENSG00000269994
C16orf95-DT	ENSG00000270006
LINC03131	ENSG00000270372
YTHDF3-DT	ENSG00000270673
CD2AP-DT	ENSG00000270761
MAP2K5-DT	ENSG00000270964
ATP2B1-AS1	ENSG00000271614
ENSG00000271855	ENSG00000271855
DCTN6-DT	ENSG00000271869
ENSG00000272092	ENSG00000272092
ENSG00000272121	ENSG00000272121
EFCAB2-AS1	ENSG00000272195
TTC23L-AS1	ENSG00000272323
CD24	ENSG00000272398
LINC01023	ENSG00000272523
ENSG00000272702	ENSG00000272702
ENSG00000272735	ENSG00000272735
ENSG00000272768	ENSG00000272768
ENSG00000273080	ENSG00000273080
ENSG00000273102	ENSG00000273102
ENSG00000273398	ENSG00000273398

CDRT15P10	ENSG00000273693
H2BC8	ENSG00000273802
ENSG00000275494	ENSG00000275494
H2BC9	ENSG00000275713
RN7SL1	ENSG00000276168
RMRP	ENSG00000277027
H2AC8	ENSG00000277075
BICRA-AS2	ENSG00000277383
H3C7	ENSG00000277775
CKAP2-DT	ENSG00000278238
ZNF426-DT	ENSG00000278611
H4C1	ENSG00000278637
H4C2	ENSG00000278705
RN7SL3	ENSG00000278771
ENSG00000278899	ENSG00000278899
ENSG00000280649	ENSG00000280649
LL0XNC01-250H12.3	ENSG00000281091
ENSG00000283103	ENSG00000283103
ENSG00000284636	ENSG00000284636
ENSG00000286282	ENSG00000286282
ENSG00000286427	ENSG00000286427
ENSG00000286527	ENSG00000286527
PARTICL	ENSG00000286532
ENSG00000287001	ENSG00000287001
ENSG00000287016	ENSG00000287016
ENSG00000287516	ENSG00000287516
ENSG00000288012	ENSG00000288012
ENSG00000288029	ENSG00000288029
ENSG00000288743	ENSG00000288743
ENSG00000288886	ENSG00000288886
ENSG00000288934	ENSG00000288934
ENSG00000288999	ENSG00000288999
ENSG00000289004	ENSG00000289004
ENSG00000289019	ENSG00000289019
ENSG00000289046	ENSG00000289046
ENSG00000289056	ENSG00000289056
ENSG00000289065	ENSG00000289065
ENSG00000289213	ENSG00000289213
ENSG00000289238	ENSG00000289238
ENSG00000289516	ENSG00000289516
ENSG00000289592	ENSG00000289592
ENSG00000290085	ENSG00000290085
ENSG00000290829	ENSG00000290829
ALOX12P2	ENSG00000291084
ENSG00000291176	ENSG00000291176
**Downregulated**	
**gene_name**	**gene_id**
CYP26B1	ENSG00000003137
TAC1	ENSG00000006128
NOS2	ENSG00000007171
TENM1	ENSG00000009694
ISL1	ENSG00000016082
PHLDB1	ENSG00000019144
MYOM2	ENSG00000036448

FAM184B	ENSG00000047662
LAMC3	ENSG00000050555
NTHL1	ENSG00000065057
SYT1	ENSG00000067715
NAV3	ENSG00000067798
SDK2	ENSG00000069188
MNT	ENSG00000070444
SLC6A15	ENSG00000072041
NOTCH3	ENSG00000074181
ZNF532	ENSG00000074657
EPHA6	ENSG00000080224
P2RX5	ENSG00000083454
ZNF671	ENSG00000083814
NLK	ENSG00000087095
RGS17	ENSG00000091844
CRAT	ENSG00000095321
SEMA4G	ENSG00000095539
NFATC4	ENSG00000100968
RENBP	ENSG00000102032
GLA	ENSG00000102393
NDRG1	ENSG00000104419
CDK6	ENSG00000105810
MEOX2	ENSG00000106511
CCND1	ENSG00000110092
MDK	ENSG00000110492
PHC1	ENSG00000111752
ULBP1	ENSG00000111981
MDN1	ENSG00000112159
STC2	ENSG00000113739
BCHE	ENSG00000114200
PDE1A	ENSG00000115252
ARID3A	ENSG00000116017
CTH	ENSG00000116761
KCNQ4	ENSG00000117013
KIF17	ENSG00000117245
SLC16A7	ENSG00000118596
HOXB5	ENSG00000120075
PLXDC2	ENSG00000120594
KCNJ8	ENSG00000121361
SLITRK3	ENSG00000121871
PTGFR	ENSG00000122420
HOXC11	ENSG00000123388
TRIP10	ENSG00000125733
FOSB	ENSG00000125740
BMP2	ENSG00000125845
DACH2	ENSG00000126733
UBR4	ENSG00000127481
CDC42EP1	ENSG00000128283
CHAC1	ENSG00000128965
SAT1	ENSG00000130066
GDF15	ENSG00000130513
NFATC1	ENSG00000131196
BARX1	ENSG00000131668
NES	ENSG00000132688

LPIN3	ENSG00000132793
IRS4	ENSG00000133124
ZFC3H1	ENSG00000133858
MSI1	ENSG00000135097
NHSL1	ENSG00000135540
ALDH1L2	ENSG00000136010
EDNRB	ENSG00000136160
MYC	ENSG00000136997
IGFBPL1	ENSG00000137142
IFI44L	ENSG00000137959
PCDH10	ENSG00000138650
FBN2	ENSG00000138829
PPFIA2	ENSG00000139220
SLC7A1	ENSG00000139514
RHOF	ENSG00000139725
TTYH2	ENSG00000141540
DMRTA2	ENSG00000142700
GALNT13	ENSG00000144278
AGTR1	ENSG00000144891
EPHA5	ENSG00000145242
SFRP2	ENSG00000145423
PLA2G7	ENSG00000146070
DOK3	ENSG00000146094
PPRC1	ENSG00000148840
SLC43A1	ENSG00000149150
VEGFC	ENSG00000150630
DCLRE1C	ENSG00000152457
ZIC1	ENSG00000152977
MERTK	ENSG00000153208
LARP1	ENSG00000155506
ADAMTS3	ENSG00000156140
TSC22D3	ENSG00000157514
C2CD2	ENSG00000157617
TENT5B	ENSG00000158246
THEM4	ENSG00000159445
CBS	ENSG00000160200
NEK8	ENSG00000160602
TLCD1	ENSG00000160606
RBP7	ENSG00000162444
ALPL	ENSG00000162551
MXRA8	ENSG00000162576
DDR2	ENSG00000162733
KCNJ3	ENSG00000162989
IFI16	ENSG00000163565
HHIP	ENSG00000164161
TERT	ENSG00000164362
CLDN3	ENSG00000165215
SLITRK5	ENSG00000165300
INTS6L	ENSG00000165359
AMN	ENSG00000166126
COL3A1	ENSG00000168542
AR	ENSG00000169083
FASN	ENSG00000169710
RAC3	ENSG00000169750

P2RY1	ENSG00000169860
MYRIP	ENSG00000170011
ZNF804A	ENSG00000170396
TRIM8	ENSG00000171206
B3GALT1	ENSG00000172318
MUS81	ENSG00000172732
SSH3	ENSG00000172830
TRIB1	ENSG00000173334
EHBP1L1	ENSG00000173442
PLK3	ENSG00000173846
TNK1	ENSG00000174292
GALNTL6	ENSG00000174473
SLC29A2	ENSG00000174669
BRSK2	ENSG00000174672
SART1	ENSG00000175467
FOSL1	ENSG00000175592
ETV4	ENSG00000175832
NECTIN3	ENSG00000177707
PLEC	ENSG00000178209
PER1	ENSG00000179094
MEIS3P1	ENSG00000179277
CHRM4	ENSG00000180720
SHISA2	ENSG00000180730
FAM83H	ENSG00000180921
RNF135	ENSG00000181481
AMIGO1	ENSG00000181754
RIPPLY3	ENSG00000183145
CTNNA3	ENSG00000183230
BCOR	ENSG00000183337
KCND2	ENSG00000184408
KRBA2	ENSG00000184619
MAFF	ENSG00000185022
PRKN	ENSG00000185345
LSAMP	ENSG00000185565
ADAMTSL5	ENSG00000185761
ARL17A	ENSG00000185829
VSTM2B	ENSG00000187135
AGMO	ENSG00000187546
PLEKHN1	ENSG00000187583
MAGEH1	ENSG00000187601
SPRY4	ENSG00000187678
TMEM220	ENSG00000187824
HACD4	ENSG00000188921
SYCP2	ENSG00000196074
S100A4	ENSG00000196154
SLC6A9	ENSG00000196517
DDI2	ENSG00000197312
PARVA	ENSG00000197702
MYO1C	ENSG00000197879
CHSY3	ENSG00000198108
ZNF849P	ENSG00000198153
ARC	ENSG00000198576
SNHG5	ENSG00000203875
COL5A2	ENSG00000204262

TAP2	ENSG00000204267
HSPA1B	ENSG00000204388
BAG6	ENSG00000204463
TRIM71	ENSG00000206557
RNU1-1	ENSG00000206652
RNVU1-18	ENSG00000206737
SH3D21	ENSG00000214193
HNRNPUL2	ENSG00000214753
MPRIPP1	ENSG00000214820
TNFRSF25	ENSG00000215788
EIF4BP7	ENSG00000225031
ENSG00000225075	ENSG00000225075
HSBP1L1	ENSG00000226742
ARL17B	ENSG00000228696
HOXB-AS3	ENSG00000233101
LINC01694	ENSG00000233922
SLC25A25-AS1	ENSG00000234771
IRS4-AS1	ENSG00000237863
RNVU1-2	ENSG00000238825
SCARF2	ENSG00000244486
USP51	ENSG00000247746
CASC11	ENSG00000249375
LINC01618	ENSG00000250302
EPHA5-AS1	ENSG00000250846
ENSG00000250899	ENSG00000250899
NIHCOLE	ENSG00000251026
ENSG00000251550	ENSG00000251550
MALAT1	ENSG00000251562
CHSY3-AS1	ENSG00000251680
LINC01605	ENSG00000253161
MEX3A	ENSG00000254726
ENSG00000254867	ENSG00000254867
ENSG00000255389	ENSG00000255389
SALL3	ENSG00000256463
LINC00648	ENSG00000259129
LINC02253	ENSG00000259485
ISL1-DT	ENSG00000259663
NSFP1	ENSG00000260075
PCDHGA9	ENSG00000261934
SNORD3B-1	ENSG00000265185
BAHCC1	ENSG00000266074
KC6	ENSG00000267313
CIRBP-AS1	ENSG00000267493
ENSG00000268573	ENSG00000268573
ENSG00000269825	ENSG00000269825
ENSG00000270022	ENSG00000270022
NBPF14	ENSG00000270629
ENSG00000272945	ENSG00000272945
ENSG00000275888	ENSG00000275888
ENSG00000277693	ENSG00000277693
ENSG00000279342	ENSG00000279342
HELLPAR	ENSG00000281344
ENSG00000283183	ENSG00000283183
CIROP	ENSG00000283654

ENSG00000283839	ENSG00000283839
ENSG00000285646	ENSG00000285646
ENSG00000286385	ENSG00000286385
ENSG00000286447	ENSG00000286447
ENSG00000286531	ENSG00000286531
ENSG00000287055	ENSG00000287055
ENSG00000287124	ENSG00000287124
TALAM1	ENSG00000289740
